# Supplementary figures and images for: Sales of antibiotics and hydroxychloroquine in India during the COVID-19 epidemic: An interrupted time series analysis
Source: PLoS Med. 2021 Jul 1;18(7):e1003682. doi: 10.1371/journal.pmed.1003682 (PMC8248656; doi:10.1371/journal.pmed.1003682)

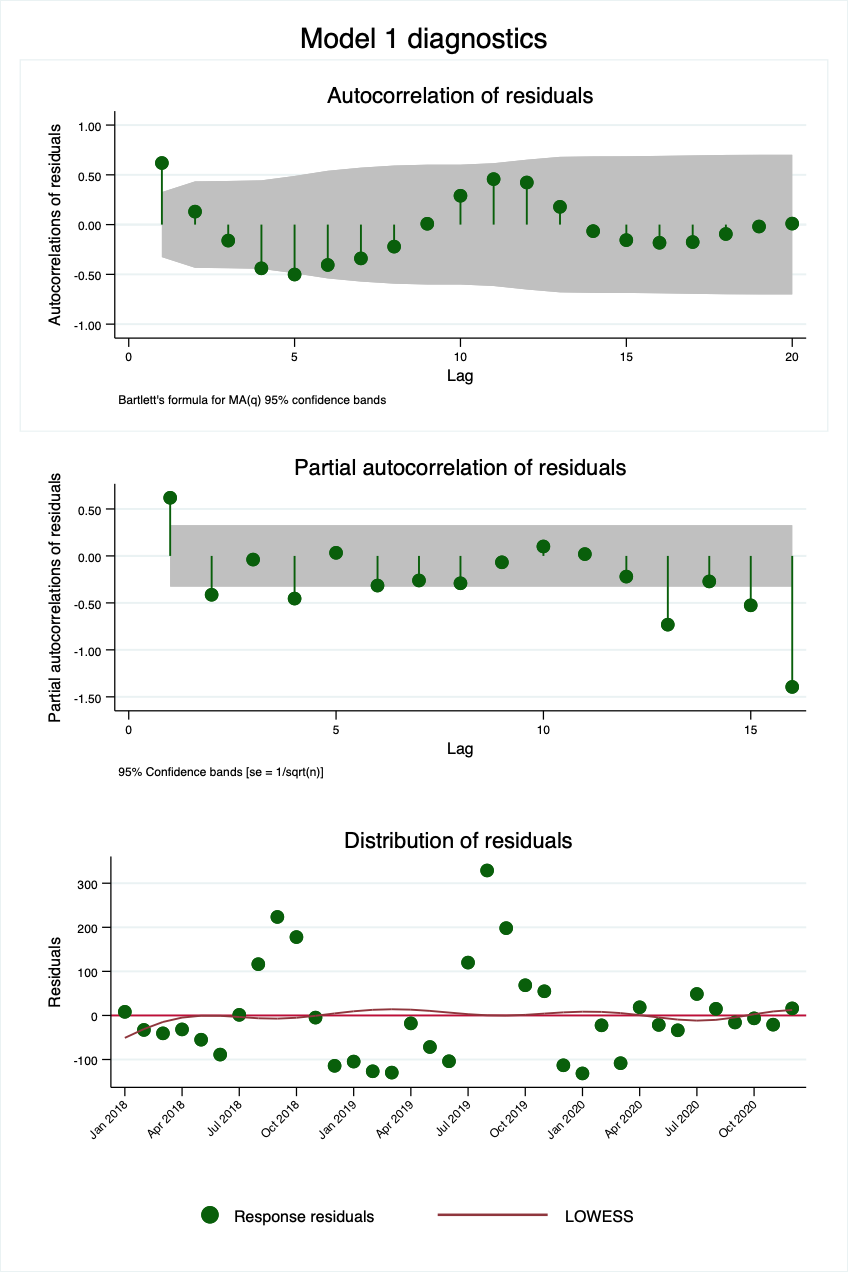

Supplement: S1 Fig — (TIF) [file pmed.1003682.s002.tif]

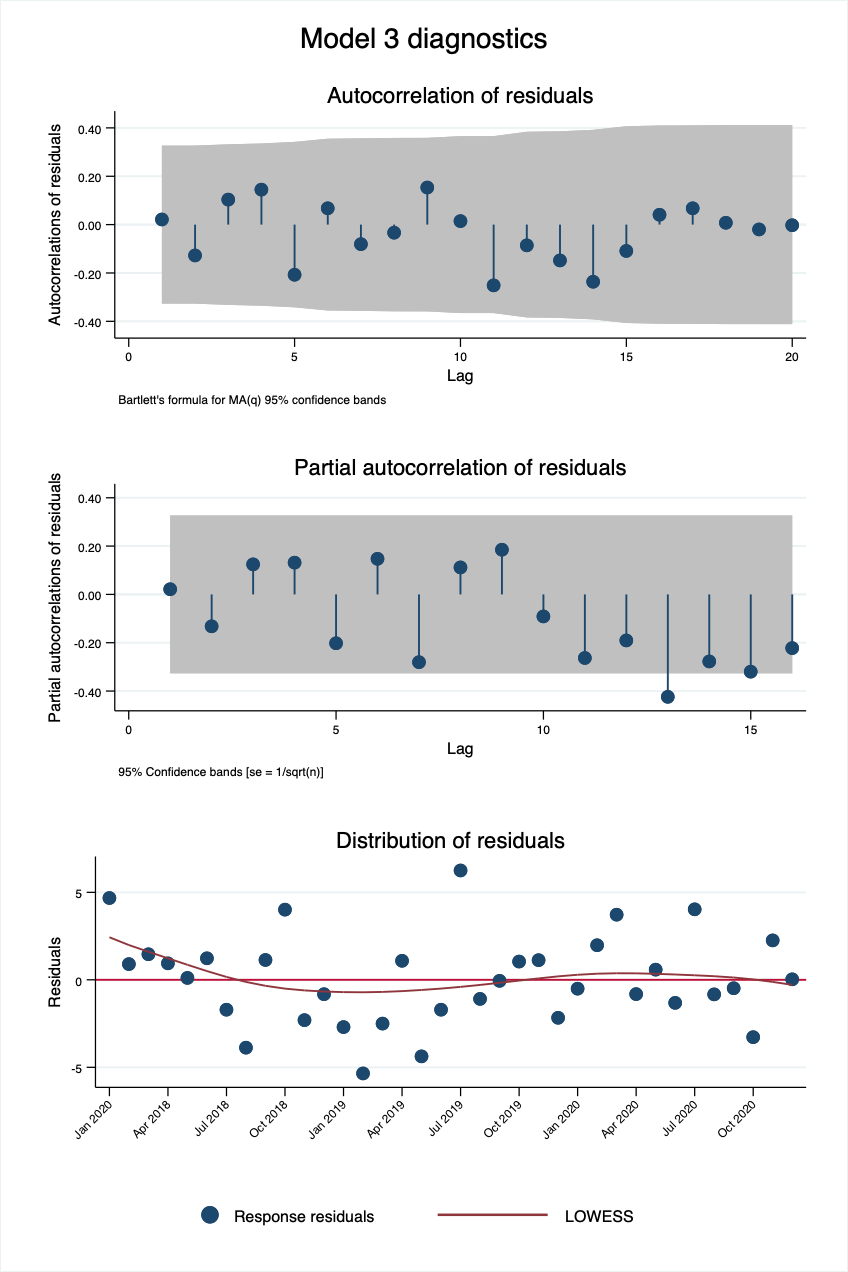

Supplement: S2 Fig — (TIF) [file pmed.1003682.s003.tif]

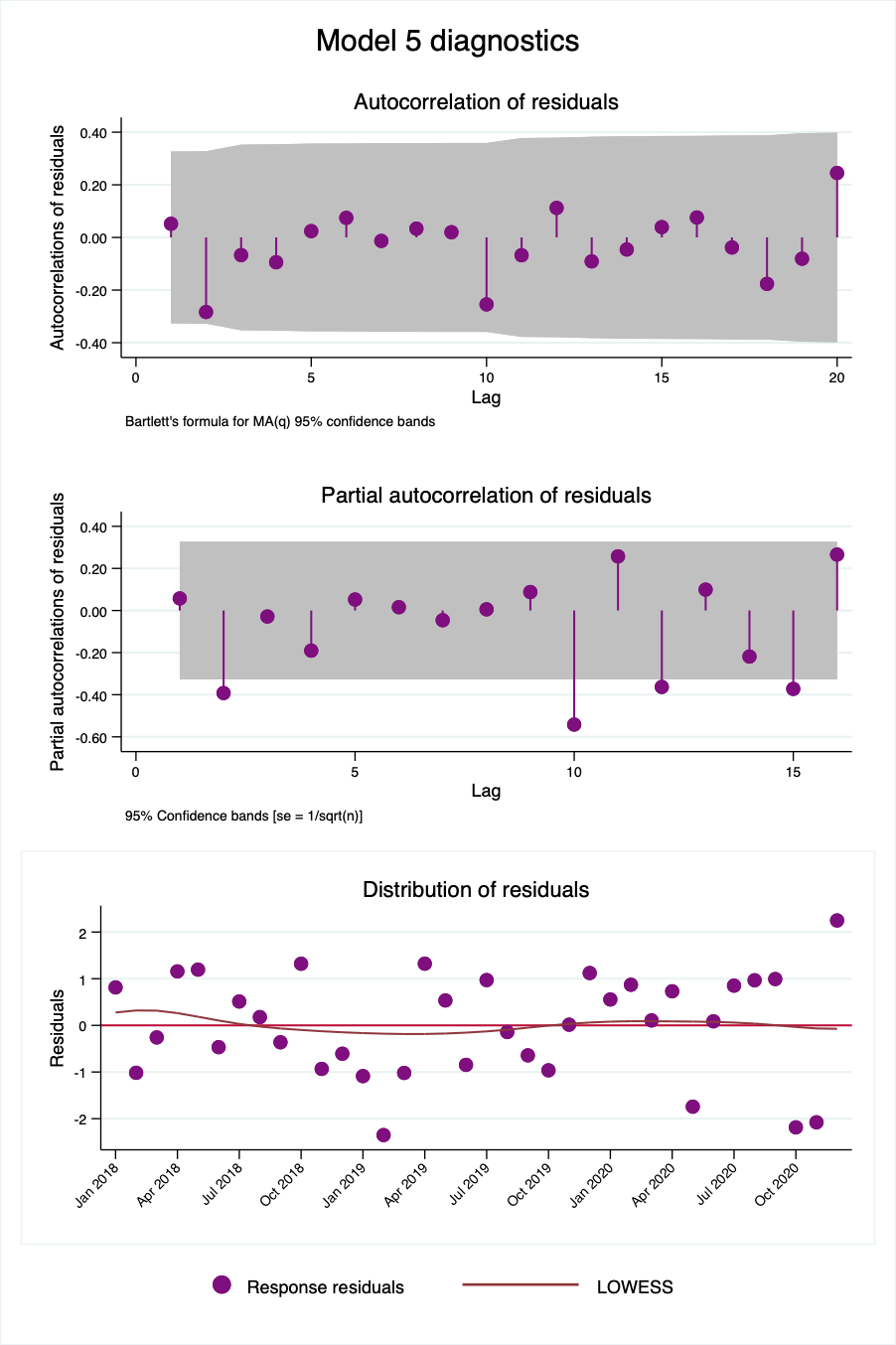

Supplement: S3 Fig — (TIF) [file pmed.1003682.s004.tif]

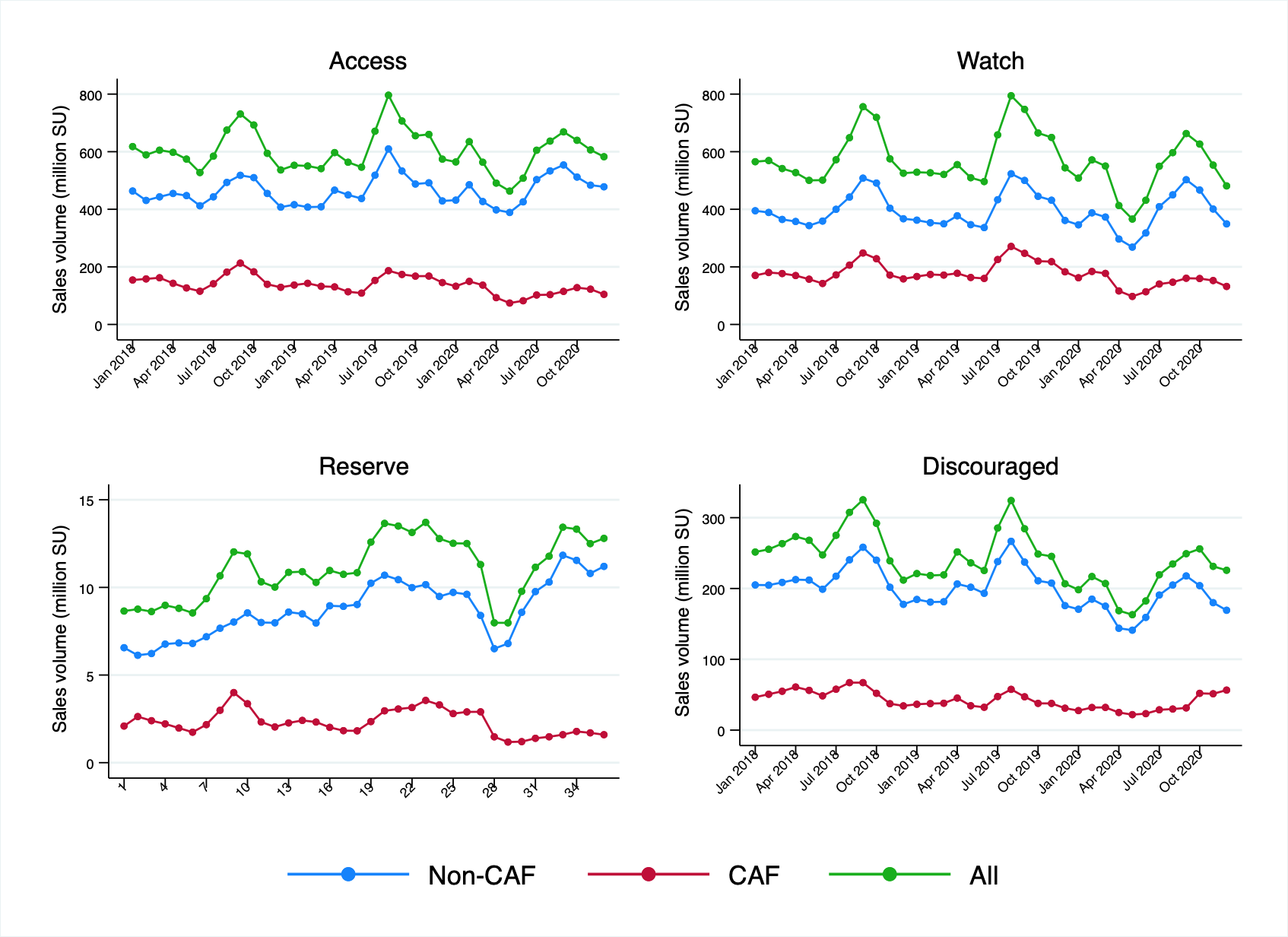

Supplement: S4 Fig — (TIF) [file pmed.1003682.s005.tif]

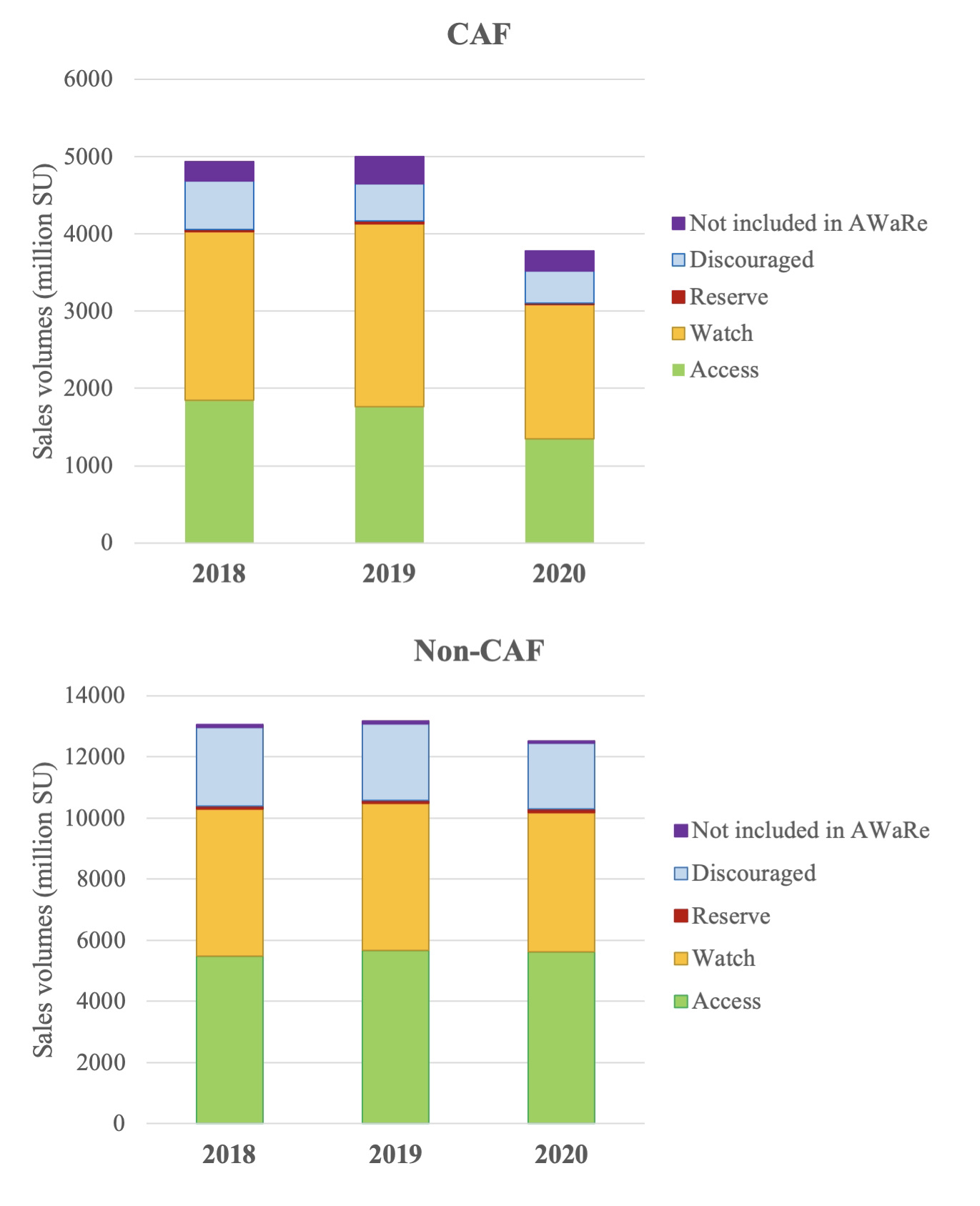

Supplement: S5 Fig — (TIF) [file pmed.1003682.s006.tif]

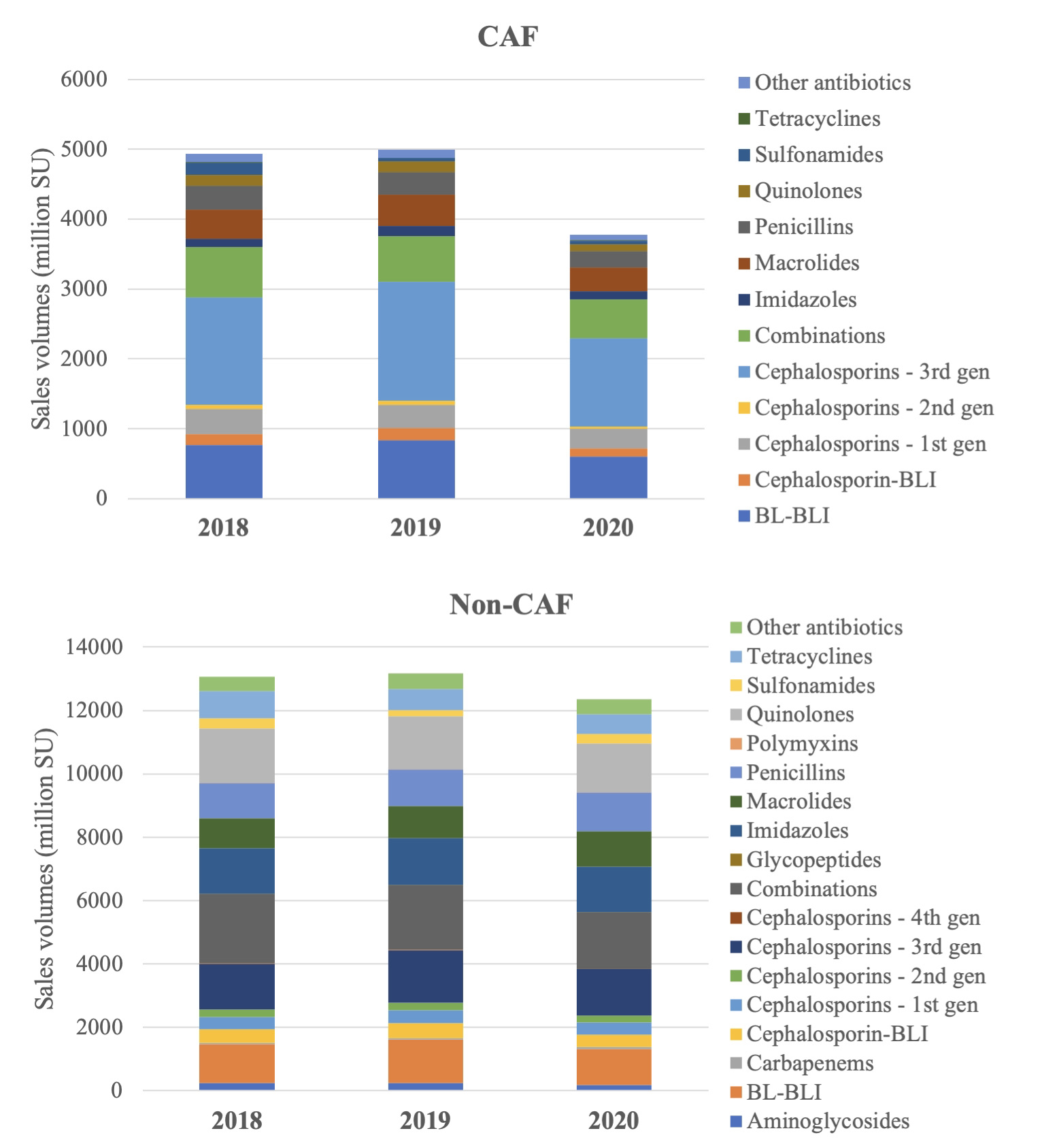

Supplement: S6 Fig — (TIF) [file pmed.1003682.s007.tif]

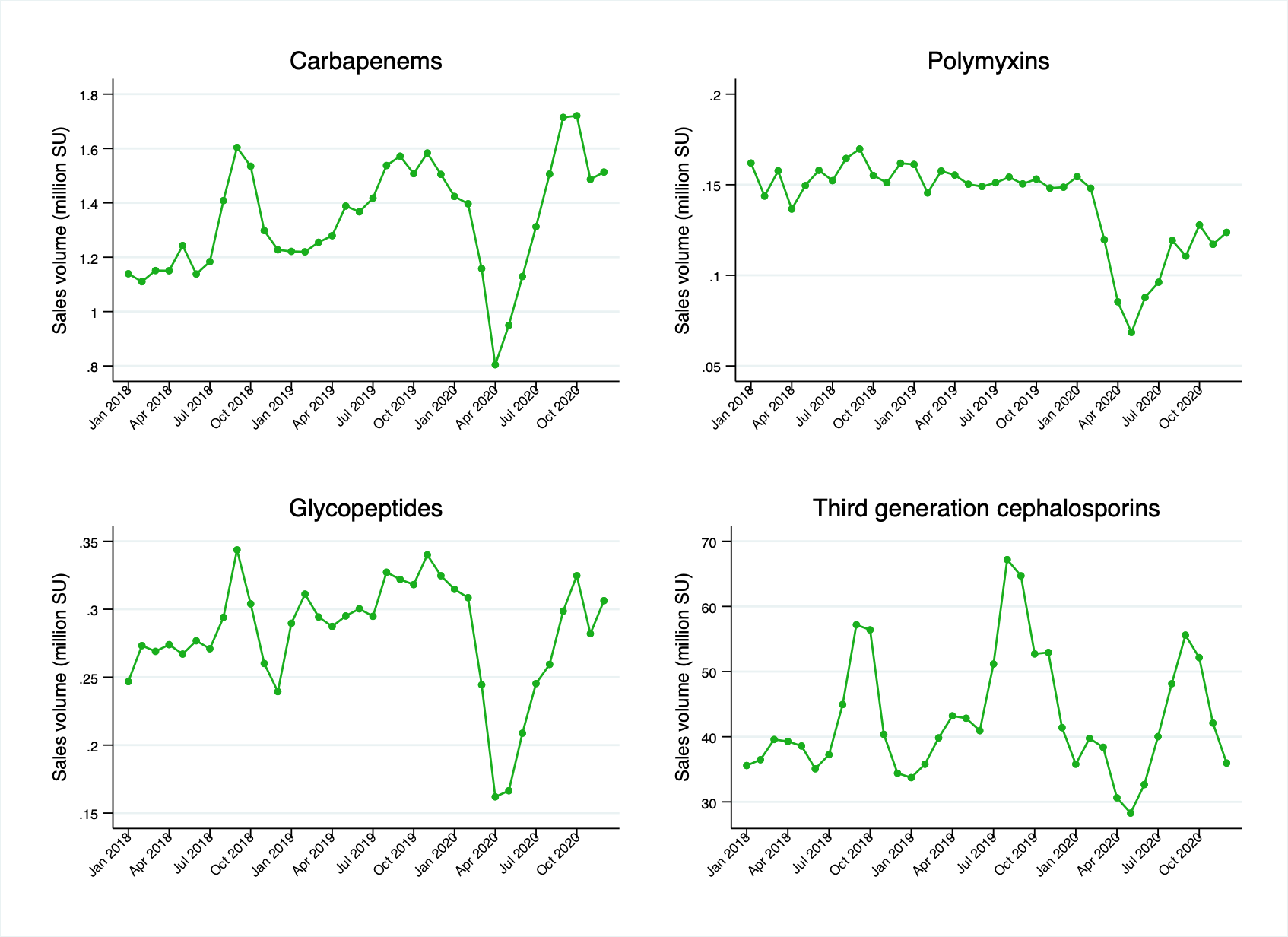

Supplement: S7 Fig — (TIF) [file pmed.1003682.s008.tif]

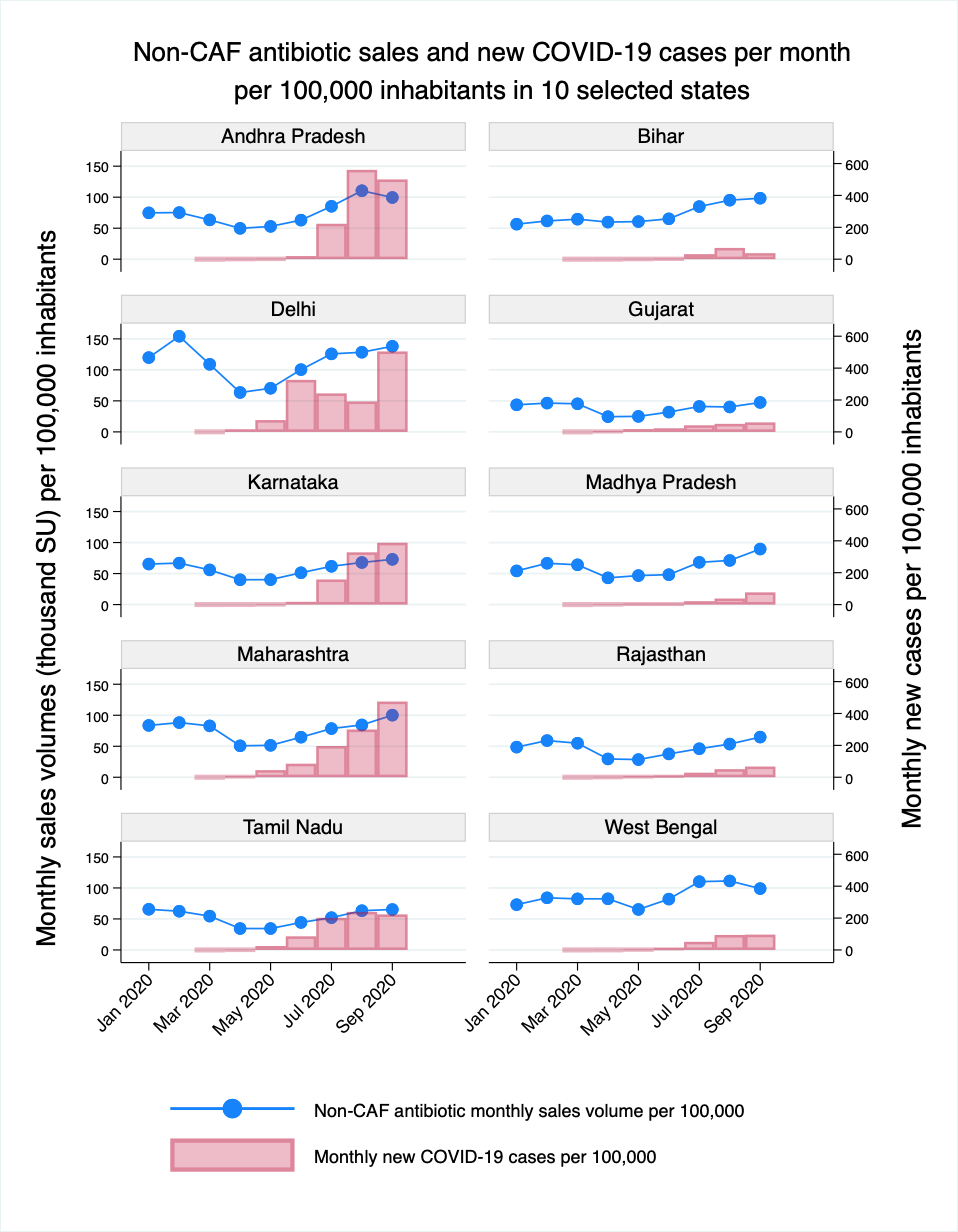

Supplement: S8 Fig — States with the highest rates of detected COVID-19 cases are shown on the left side of the graph, whereas states with the lowest rates of detected COVID-19 cases are on the right. (TIF) [file pmed.1003682.s009.tif]

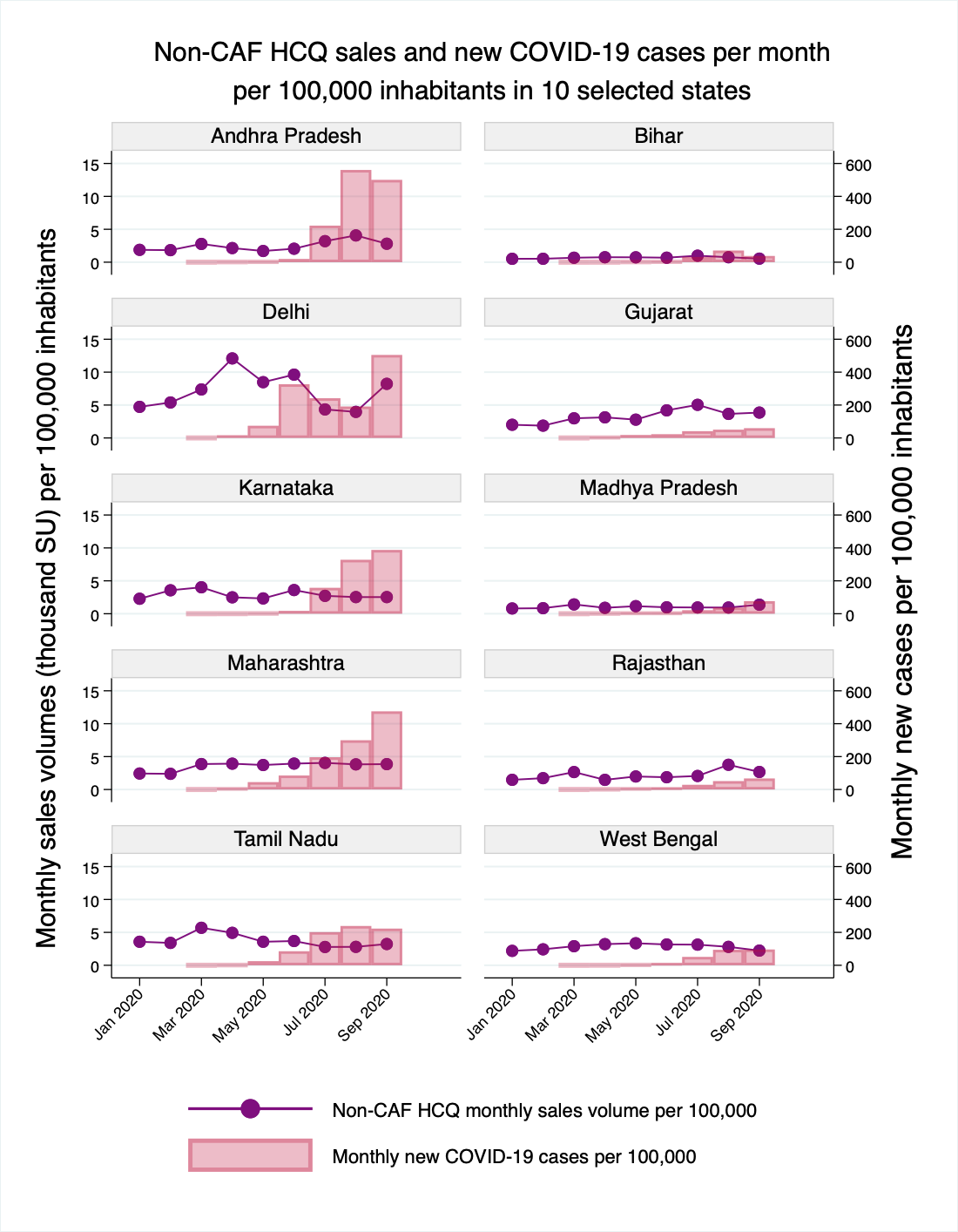

Supplement: S9 Fig — States with the highest rates of detected COVID-19 cases are shown on the left side of the graph, whereas states with the lowest rates of detected COVID-19 cases are on the right. (TIF) [file pmed.1003682.s010.tif]

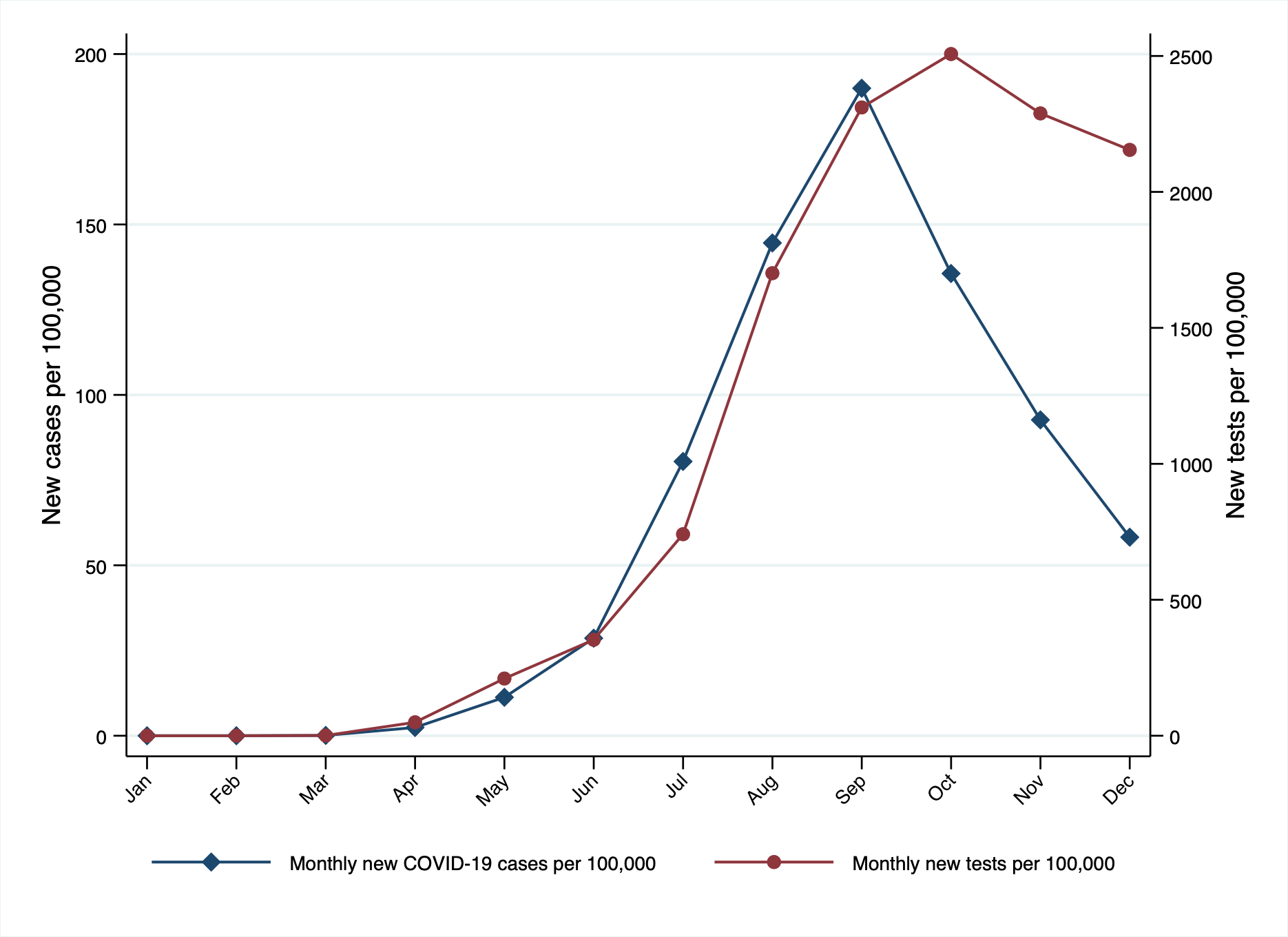

Supplement: S10 Fig — (TIF) [file pmed.1003682.s011.tif]
