# Supplementary material for: Sales of antibiotics and hydroxychloroquine in India during the COVID-19 epidemic: An interrupted time series analysis
Source: PLoS Med. 2021 Jul 1;18(7):e1003682. doi: 10.1371/journal.pmed.1003682 (PMC8248656; doi:10.1371/journal.pmed.1003682)
Supplement: S1 Table — (PDF) [file pmed.1003682.s012.pdf]

S1 Table: Search strategy used in the rapid systematic review regarding the impact of COVID-19 pandemic on antibiotic use.

Concept	Search terms
COVID-19	(("Severe acute respiratory syndrome coronavirus 2"[nm] OR "COVID-19"[nm] OR 2019-nCoV[tiab] OR 2019nCoV[tiab] OR COVID-19[tiab] OR COVID19[tiab] OR SARS-CoV-2[tiab] OR SARS COV2[tiab] OR SARSCOV2[tiab] OR SARSCOV 2[tiab] OR (((wuhan[all fields] AND coronavirus*[tiab]) OR new coronavirus[tiab] OR novel coronavirus[tiab]))
AND	
Antibiotic	(("anti-bacterial agents"[Pharmacological Action] OR "anti-bacterial agents"[MeSH Terms] OR "anti-infective agents"[Pharmacological Action] OR "anti-infective agents"[MeSH Terms] OR antibiotic*[tw] OR antimicrobial*[tw] OR antibacterial*[tw] OR anti bacterial*[tw] OR anti-infective*[tw]))
