# Supplementary material for: Sales of antibiotics and hydroxychloroquine in India during the COVID-19 epidemic: An interrupted time series analysis
Source: PLoS Med. 2021 Jul 1;18(7):e1003682. doi: 10.1371/journal.pmed.1003682 (PMC8248656; doi:10.1371/journal.pmed.1003682)
Supplement: S2 Table — (PDF) [file pmed.1003682.s013.pdf]

S2 Table: Features and findings of studies that evaluated the impact of COVID-19 on antibiotic use.

Study reference	Country	Design	Population and setting	Main findings
Abelenda-Alonso et al, *Infect Contr Hosp Epidemiol* (2020) [1]	Spain	Before-and-after cross-sectional study comparing antibiotic dispensing in Jan-Apr 2019 versus Jan-Apr 2020.	All patients admitted to Bellvitge University Hospital (Barcelona).	▪ Similar levels of antibiotic use in Jan-Feb 2019 versus 2020.<br>▪ Significant increase in dispensing in Mar-Apr 2020 compared to 2019 ($p<0.001$).<br>▪ Rapid increase in use of amoxicillin/clavulanate in Mar 2020, followed by an increase in broad-spectrum agents in April.
Buehrle et al, *Antimicrob Agents Chemother* (2020) [2]	United States	Interrupted time series analysis of selected antibiotic use between Jan 2018 and June 2020, comparing pre-pandemic period versus pandemic period (from March 2020).	All patients admitted at VA Pittsburgh hospital.	▪ 6.5% (95% CI: 3.0-10.1) monthly reduction in antibiotic use in Mar-Jun 2020 versus Jan 2018 – Feb 2020.<br>▪ 1.3% (95% CI: 0.7-4.8) monthly increase in antibiotic DOT per 1.000 patient bed days of care in Mar-Jun 2020.<br>▪ Significant increases in use of non-antipseudomonal penicillins and macrolides, with decrease in use of antipseudomonal penicillins, non-antipseudomonal cephalosporins and quinolones.
Dieringer et al, *Infect Control Hosp Epidemiol* (2020) [3]	United States	Before-and-after study comparing antibiotic use in Jan-May 2020 versus the same period of 2015-2019.	All patients admitted to acute inpatient care in 84 facilities of Veterans' Health Administration.	▪ In Jan-May of each year during 2015-2019, antibiotic use decreased from 638 to 602 DOT per 1,000 DP (mean decrease, 9.1 DOT per 1,000 DP per year).<br>▪ Antibiotic use increased from 602 to 628 DOT per 1,000 DP in Jan-May 2020.<br>▪ Greatest increase in broad-spectrum agents used to treat community-acquired and hospital-acquired infections.
Gonzalez-Zorn, *Clin Microbiol Infect* (2020) [4]	Spain	Descriptive study of antibiotic use in Spain based on time series data from IQVIA comparing March 2020 against the period Jan 2017-Feb 2020.	National antibiotic sales data, presumably in the private sector only.	▪ Azithromycin use in March 2020 was 400% the use in February 2020 and 320% that of January 2019.<br>▪ Other antibiotics increased in consumption in March 2020 as compared to February 2020 (e.g. ceftaroline, ceftolozane/tazobactam, cefditoren, ceftriaxone, colistin, doxycycline and linezolid).<br>▪ No impact evaluation and no adjustment for seasonality were performed.
Katz et al, *J Ped Infect Dis Soc* (2020) [5]	United States	Before-and-after study comparing ambulatory pediatric antibiotic prescription rates and diagnoses in Mar-May 2020 versus Mar-May 2019.	Prescription data from 4 ambulatory settings affiliated with Vanderbilt University Medical Center (i.e. emergency department, urgent care clinics, primary care clinics, and retail health clinics).	▪ The number of visits and the proportion of visits for infectious conditions declined in 2020 (4267/7010 [60.8%] vs 11 412/16 671 [68.5%] in 2019; $P < 0.001$).<br>▪ The percent of visits with an antibiotic prescription was lower in 2020 vs 2019 both overall (2240/7010 [32%] vs 6373/16 671 [38.2%], $P < 0.001$) and among visits for infectious diseases (1324/2943 [45%] vs 3941/7471 [52.8%], $P < 0.001$).
Nestler et al, *Infect Contr Hosp Epidemiol* (2020) [6]	United States	Before-and-after study comparing selected antibiotic use in Apr 2019 – Mar 2020 versus Apr-May 2020.	Pneumonia patients admitted to MICU, CICU or PM unit at Virginia Commonwealth University (VCU) Health System, an 865-bed urban academic medical center.	▪ Significant increase in ceftriaxone (+131, +138 and +193 DOT per 1,000 patient-days respectively in CICU, PM and MICU) and azithromycin (+103 and +109 DOT per 1,000 patient-days respectively in PM and MICU) use in Apr 2020.<br>▪ Significant decrease in levofloxacin use in May 2020 (-14 and -24 DOT per 1,000 patient-days respectively in CICU and MICU).

Study reference	Country	Design	Population and setting	Main findings
Staub et al, *Infect Contr Hosp Epidemiol* (2020) [7]	United States	Before-and-after study comparing antibiotic use across three periods: Dec 2019 – Feb 2020, Mar 2020 and Apr-May 2020.	Patients admitted to either IM or MICU at Vanderbilt University Medical Center.	▪ Increase in weekly antibiotic use in the first COVID-19 period versus pre-COVID-19, both in IM (+145.3 DOT/1,000 days) and MICU (+204 DOT/1,000 days).<br>▪ Significant decrease in weekly azithromycin use (-58.2 DOT/1,000 days) during the second COVID-19 period in IM (no change in MICU).
Velasco-Arnaiz et al, *Infect Contr Hosp Epidemiol* (2020) [8]	Spain	Before-and after study comparing antibiotic use in Feb-Apr 2020 versus the same period of 2019.	Patients admitted to PICU and non-PICU areas at San Joan de Deu Hospital, Barcelona.	▪ Increase in total antibiotic use in Mar 2020 versus Mar 2019 (+1.6 DOT/100 DP, mainly in non-PICU) and in Apr 2020 versus Apr 2019 (+35.5 DOT/100 DP, mainly in PICU).<br>▪ Increase in azithromycin use in 2020 versus 2019, mostly associated with hydroxychloroquine, particularly in PICU.

Abbreviations: CICU, coronary intensive care unit; DOT, days of therapy; DP, days present; IM, internal medicine; MICU, medical intensive care unit; PICU, pediatric intensive care unit; PM, progressive medicine.

References

1. Abelenda-Alonso G, Padullés A, Rombauts A, Gudiol C, Pujol M, Alvarez-Pouso C, et al. Antibiotic prescription during the COVID-19 pandemic: A biphasic pattern. Infect Control Hosp Epidemiol. 2020;41(11):1371-2. Epub 2020/07/31. doi: 10.1017/ice.2020.381. PubMed PMID: 32729437; PubMed Central PMCID: PMCPMC7426604.
2. Buehrle DJ, Decker BK, Wagener MM, Adalja A, Singh N, McEllistrem MC, et al. Antibiotic Consumption and Stewardship at a Hospital outside of an Early Coronavirus Disease 2019 Epicenter. Antimicrob Agents Chemother. 2020;64(11). Epub 2020/08/21. doi: 10.1128/aac.01011-20. PubMed PMID: 32816693; PubMed Central PMCID: PMCPMC7577150.
3. Dieringer TD, Furukawa D, Graber CJ, Stevens VW, Jones MM, Rubin M, et al. Inpatient antibiotic utilization in the Veterans Administration during the COVID-19 pandemic. Infect Control Hosp Epidemiol. 2020:1-9. Epub 2020/10/21. doi: 10.1017/ice.2020.1277. PubMed PMID: 33077000.
4. Gonzalez-Zorn B. Antibiotic use in the COVID-19 crisis in Spain. Clin Microbiol Infect. 2020. Epub 2020/11/30. doi: 10.1016/j.cmi.2020.09.055. PubMed PMID: 33248926; PubMed Central PMCID: PMCPMC7688281.
5. Katz SE, Spencer H, Zhang M, Banerjee R. Impact of the COVID-19 Pandemic on Infectious Diagnoses and Antibiotic Use in Pediatric Ambulatory Practices. Journal of the Pediatric Infectious Diseases Society. 2020. doi: 10.1093/jpids/piaa124.
6. Nestler M, Godbout E, Lee K, Kim J, Noda AJ, Taylor P, et al. Impact of COVID-19 on Pneumonia-Focused Antibiotic Use at an Academic Medical Center. Infect Control Hosp Epidemiol. 2020:1-9. Epub 2020/07/24. doi: 10.1017/ice.2020.362. PubMed PMID: 32698920.

7. Staub MB, Beaulieu RM, Graves J, Nelson GE. Changes in Antimicrobial Utilization During the COVID-19 Pandemic after Implementation of a Multispecialty Clinical Guidance Team. Infect Control Hosp Epidemiol. 2020:1-28. Epub 2020/10/27. doi: 10.1017/ice.2020.1291. PubMed PMID: 33100250.
8. Velasco-Arnaiz E, López-Ramos MG, Simó-Nebot S, Jordan I, Ríos-Barnés M, Urrea-Ayala M, et al. Pediatric antimicrobial stewardship in the COVID-19 outbreak. Infect Control Hosp Epidemiol. 2020:1-3. Epub 2020/06/25. doi: 10.1017/ice.2020.312. PubMed PMID: 32576298; PubMed Central PMCID: PMCPMC7338437.
