# Supplementary material for: Sales of antibiotics and hydroxychloroquine in India during the COVID-19 epidemic: An interrupted time series analysis
Source: PLoS Med. 2021 Jul 1;18(7):e1003682. doi: 10.1371/journal.pmed.1003682 (PMC8248656; doi:10.1371/journal.pmed.1003682)
Supplement: S3 Table — (PDF) [file pmed.1003682.s014.pdf]

S3 Table: List of oral formulations considered as child-appropriate

**Type**	**Formulation**
Solid	Chewable tablets
	Dispersible tablets
	"Kid" tablets*
	"Paediatric" tablets*
Liquid	Drops
	Dry suspensions
	Ordinary liquids
	Soluble powders
	Syrups

* According to the package label
