# Supplementary material for: Sales of antibiotics and hydroxychloroquine in India during the COVID-19 epidemic: An interrupted time series analysis
Source: PLoS Med. 2021 Jul 1;18(7):e1003682. doi: 10.1371/journal.pmed.1003682 (PMC8248656; doi:10.1371/journal.pmed.1003682)
Supplement: S4 Table — (PDF) [file pmed.1003682.s015.pdf]

S4 Table: List of all antibiotics included in our dataset, along with class, AWaRe category (2019) and Schedule H/H1.

Molecule	AWaRe category	Class	Schedule
Amikacin	Access	Aminoglycosides	H
Amikacin/Cefepime	Discouraged	Combinations	H1
Amoxicillin	Access	Penicillins	H
Amoxicillin/Clavulanate	Access	BL-BLI	H
Amoxicillin/Cloxacillin	Discouraged	Combinations	H
Amoxicillin/Dicloxacillin	Discouraged	Combinations	H
Amoxicillin/Flucloxacillin	Discouraged	Combinations	H
Amoxicillin/Sulbactam	Discouraged	BL-BLI	H
Amoxicillin/Tinidazole	Not included	Combinations	H
Ampicillin	Access	Penicillins	H
Ampicillin/Cloxacillin	Discouraged	Combinations	H
Ampicillin/Dicloxacillin	Discouraged	Combinations	H
Ampicillin/Flucloxacillin	Discouraged	Combinations	H
Ampicillin/Sulbactam	Access	BL-BLI	H
Arbekacin	Watch	Aminoglycosides	H
Azithromycin	Watch	Macrolides	H
Azithromycin/Cefixime	Discouraged	Combinations	H1
Azithromycin/Cefpodoxime	Discouraged	Combinations	H1
Azithromycin/Fluconazole/Ornidazole	Not included	Combinations	H
Azithromycin/Levofloxacin	Discouraged	Combinations	H1
Azithromycin/Ofloxacin	Discouraged	Combinations	H
Aztreonam	Reserve	Other antibiotics	H
Balofloxacin	Watch	Quinolones	H
Cefaclor	Watch	Cephalosporins - 2nd	H
Cefadroxil	Access	Cephalosporins - 1st	H
Cefadroxil/Clavulanate	Discouraged	Cephalosporin - BLI	H
Cefalexin	Access	Cephalosporins - 1st	H
Cefalexin/Clavulanate	Discouraged	Cephalosporin - BLI	H
Cefazolin	Access	Cephalosporins - 1st	H
Cefdinir	Watch	Cephalosporins - 3rd	H1
Cefditoren	Watch	Cephalosporins - 3rd	H
Cefepime	Watch	Cephalosporins - 4th & higher	H1
Cefepime/Sulbactam	Discouraged	Cephalosporin - BLI	H1
Cefepime/Tazobactam	Discouraged	Cephalosporin - BLI	H1
Cefetamet	Watch	Cephalosporins - 3rd	H1
Cefixime	Watch	Cephalosporins - 3rd	H1
Cefixime/Cefpodoxime	Discouraged	Combinations	H1
Cefixime/Clavulanate	Discouraged	Cephalosporin - BLI	H1

Molecule	AWaRe category	Class	Schedule
Cefixime/Cloxacillin	Discouraged	Combinations	H1
Cefixime/Dicloxacillin	Discouraged	Combinations	H1
Cefixime/Levofloxacin	Discouraged	Combinations	H1
Cefixime/Linezolid	Discouraged	Combinations	H1
Cefixime/Moxifloxacin	Discouraged	Combinations	H1
Cefixime/Ofloxacin	Discouraged	Combinations	H1
Cefixime/Ornidazole	Discouraged	Combinations	H1
Cefixime/Sulbactam	Discouraged	Cephalosporin - BLI	H1
Cefoperazone	Watch	Cephalosporins - 3rd	H1
Cefoperazone/Sulbactam	Discouraged	Cephalosporin - BLI	H1
Cefoperazone/Tazobactam	Discouraged	Cephalosporin - BLI	H1
Cefotaxime	Watch	Cephalosporins - 3rd	H1
Cefotaxime/Sulbactam	Discouraged	Cephalosporin - BLI	H1
Cefpirome	Reserve	Cephalosporins - 4th & higher	H1
Cefpirome/Sulbactam	Discouraged	Cephalosporin - BLI	H1
Cefpodoxime	Watch	Cephalosporins - 3rd	H1
Cefpodoxime/Clavulanate	Discouraged	Cephalosporin - BLI	H1
Cefpodoxime/Cloxacillin	Discouraged	Combinations	H1
Cefpodoxime/Dicloxacillin	Discouraged	Combinations	H1
Cefpodoxime/Levofloxacin	Discouraged	Combinations	H1
Cefpodoxime/Ofloxacin	Discouraged	Combinations	H1
Cefpodoxime/Sulbactam	Discouraged	Cephalosporin - BLI	H1
Cefprozil	Watch	Cephalosporins - 2nd	H
Ceftaroline	Reserve	Cephalosporins - 4th & higher	H
Ceftazidime	Watch	Cephalosporins - 3rd	H1
Ceftazidime/Avibactam	Reserve	Cephalosporin - BLI	H1
Ceftazidime/Sulbactam	Discouraged	Cephalosporin - BLI	H1
Ceftazidime/Tazobactam	Discouraged	Cephalosporin - BLI	H1
Ceftazidime/Tobramycin	Discouraged	Combinations	H1
Ceftizoxime	Watch	Cephalosporins - 3rd	H1
Ceftizoxime/Sulbactam	Discouraged	Cephalosporin - BLI	H1
Ceftizoxime/Tazobactam	Discouraged	Cephalosporin - BLI	H1
Ceftriaxone	Watch	Cephalosporins - 3rd	H1
Ceftriaxone/Sulbactam	Discouraged	Cephalosporin - BLI	H1
Ceftriaxone/Tazobactam	Discouraged	Cephalosporin - BLI	H1
Ceftriaxone/Vancomycin	Discouraged	Combinations	H1
Cefuroxime	Watch	Cephalosporins - 2nd	H
Cefuroxime/Clavulanate	Discouraged	Cephalosporin - BLI	H
Cefuroxime/Linezolid	Discouraged	Combinations	H

Molecule	AWaRe category	Class	Schedule
Cefuroxime/Ornidazole	Not included	Combinations	H
Cefuroxime/Sulbactam	Discouraged	Cephalosporin - BLI	H
Chloramphenicol	Access	Other antibiotics	H
Ciprofloxacin	Watch	Quinolones	H
Ciprofloxacin/Metronidazole	Discouraged	Combinations	H
Ciprofloxacin/Ornidazole	Discouraged	Combinations	H
Ciprofloxacin/Tinidazole	Discouraged	Combinations	H
Clarithromycin	Watch	Macrolides	H
Clarithromycin/Tinidazole	Not included	Combinations	H
Clindamycin	Access	Other antibiotics	H
Cloxacillin	Access	Penicillins	H
Colistin	Reserve	Polymyxins	H
Daptomycin	Reserve	Other antibiotics	H
Dicloxacillin	Access	Penicillins	H
Diloxanide/Metronidazole	Not included	Combinations	H
Diloxanide/Tinidazole	Not included	Combinations	H
Doripenem	Watch	Carbapenems	H1
Doxycycline	Access	Tetracyclines	H
Doxycycline/Ornidazole	Not included	Combinations	H
Doxycycline/Tinidazole	Discouraged	Combinations	H
Ertapenem	Watch	Carbapenems	H1
Erythromycin	Watch	Macrolides	H
Faropenem	Reserve	Carbapenems	H1
Flucloxacillin	Access	Penicillins	H
Fluconazole/Ornidazole	Not included	Combinations	H
Fluconazole/Tinidazole	Not included	Combinations	H
Fosfomycin (O)	Watch	Other antibiotics	H
Fosfomycin (P)	Reserve	Other antibiotics	H
Furazolidone/Metronidazole	Not included	Combinations	H
Garenoxacin	Watch	Quinolones	H
Gatifloxacin	Watch	Quinolones	H
Gatifloxacin/Ornidazole	Discouraged	Combinations	H
Gemifloxacin	Watch	Quinolones	H1
Gentamicin	Access	Aminoglycosides	H
Imipenem/Cilastatin	Watch	Carbapenems	H1
Isepamicin	Watch	Aminoglycosides	H
Kanamycin	Watch	Aminoglycosides	H
Levofloxacin	Watch	Quinolones	H1
Levofloxacin/Metronidazole	Discouraged	Combinations	H1

Molecule	AWaRe category	Class	Schedule
Levofloxacin/Ornidazole	Discouraged	Combinations	H1
Lincomycin	Watch	Other antibiotics	H
Linezolid	Reserve	Other antibiotics	H
Lomefloxacin	Watch	Quinolones	H
Lymecycline	Watch	Tetracyclines	H
Meropenem	Watch	Carbapenems	H1
Meropenem/Sulbactam	Discouraged	Carbapenem - BLI	H1
Meropenem/Tazobactam	Discouraged	Carbapenem - BLI	H1
Metronidazole	Access	Imidazoles	H
Metronidazole/Nalidixic Acid	Not included	Combinations	H
Minocycline (O)	Watch	Tetracyclines	H
Minocycline (P)	Reserve	Tetracyclines	H
Moxifloxacin	Watch	Quinolones	H1
Nalidixic acid	Watch	Quinolones	H
Netilmicin	Watch	Aminoglycosides	H
Nimorazole/Ofloxacin	Discouraged	Combinations	H
Nitrofurantoin	Access	Other antibiotics	H
Norfloxacin	Watch	Quinolones	H
Norfloxacin/Metronidazole	Discouraged	Combinations	H
Norfloxacin/Tinidazole	Discouraged	Combinations	H
Ofloxacin	Watch	Quinolones	H
Ofloxacin/Metronidazole	Not included	Combinations	H
Ofloxacin/Ornidazole	Discouraged	Combinations	H
Ofloxacin/Tinidazole	Discouraged	Combinations	H
Ornidazole	Not included	Imidazoles	H
Oxytetracycline	Watch	Tetracyclines	H
Pazufloxacin	Watch	Quinolones	H
Penicillin G	Access	Penicillins	H
Penicillin G/Streptomycin	Discouraged	Combinations	H
Penicillin V	Access	Penicillins	H
Piperacillin/Tazobactam	Watch	BL-BLI	H
Polymyxin B	Reserve	Polymyxins	H
Prulifloxacin	Watch	Quinolones	H1
Roxithromycin	Watch	Macrolides	H
Sparfloxacin	Watch	Quinolones	H1
Spiramycin	Watch	Macrolides	H
Streptomycin	Watch	Aminoglycosides	H
Sulbactam	Not included	BLI	H
Sultamicillin	Access	Penicillins	H

Molecule	AWaRe category	Class	Schedule
Teicoplanin	Watch	Glycopeptides	H
Tetracycline	Access	Tetracyclines	H
Tetracycline/Tinidazole	Not included	Combinations	H
Ticarcillin/Clavulanate	Discouraged	BL-BLI	H
Tigecycline	Reserve	Other antibiotics	H
Tinidazole	Not included	Imidazoles	H
Tobramycin	Watch	Aminoglycosides	H
Trimethoprim	Access	Sulfonamides	H
Trimethoprim/Sulfamethoxazole	Access	Sulfonamides	H
Vancomycin	Watch	Glycopeptides	H

Abbreviations: Access Watch Reserve; BL, beta-lactam; BLI, beta-lactamase inhibitor
