# Supplementary material for: Sales of antibiotics and hydroxychloroquine in India during the COVID-19 epidemic: An interrupted time series analysis
Source: PLoS Med. 2021 Jul 1;18(7):e1003682. doi: 10.1371/journal.pmed.1003682 (PMC8248656; doi:10.1371/journal.pmed.1003682)
Supplement: S1 Text — (PDF) [file pmed.1003682.s016.pdf]

S1 Text: Summary of the evidence regarding the impact of COVID-19 pandemic on antibiotic use.

Objective

We conducted a rapid systematic review to collate the available evidence on the impact of COVID-19 on antimicrobial utilization.

Methods

We searched PubMed for potentially relevant studies published from December 31st to present, using a combination of terms related to the concepts of "COVID-19" and "antibiotic" (**S1 Table**). No restrictions were placed with regards to language, geographic area or population. The screening process was performed in two steps (title/abstract screening followed by full-text screening) in order to select studies that had evaluated antibiotic consumption trends prior to and during the pandemic. Studies aimed at assessing the efficacy or effectiveness of specific therapeutic regimens for COVID-19 cases and those solely reporting on the proportion of COVID-19 patients receiving antimicrobials without any sort of comparison against the pre-pandemic period were considered ineligible. Qualitative studies that reported on prescribing behaviours among practitioners were also excluded, unless quantitative data on actual antibiotic use were also collected.

Key findings

A total of 3,553 unique citations were identified as of December 2nd, 2020. After title and abstract screening, 52 publications were deemed potentially relevant and were thus selected for full-text evaluation. We excluded:

- 22 studies that only reported on the proportion of bacterial coinfections among COVID-19 cases and/or the proportion of these patients receiving antimicrobial treatment without attempting any kind of comparison with the pre-pandemic phase [1-22];
- 15 commentaries or perspective pieces [23-37];
- 7 reviews (including two systematic reviews on the incidence of bacterial co-infections in COVID-19 cases) [38-44].

We found 8 ecologic studies aimed at assessing the impact of the pandemic on antibiotic use, conducted either in Spain (3/8) or in the United States (5/8) (**S2 Table**) [45-52]. All but one were hospital-based studies, the exception being a study based on IQVIA data from Spain which did not distinguish between inpatient and outpatient care. Most of these studies (6/8) utilized a difference-in-difference analysis technique to compare antibiotic use levels between two or more periods before and during the pandemic, and one study used an interrupted time series analysis to examine the trends in consumption over time and evaluate the impact of COVID-19 from March 2020 onwards [46]. The study based on IQVIA data only provided a descriptive analysis of time series data from 2017 to March 2020 [51]. Although populations and settings varied substantially across studies, a consistent increase in antibiotic use in inpatient adult care was observed particularly in March and April 2020. This increase was mostly attributable to a greater use of selected antibiotics such as azithromycin and, in certain settings, ceftriaxone. In contrast, antibiotic use was reported to be lower in pediatric care settings [52], possibly reflecting the lower number of visits occurred during the pandemic period as compared to the pre-pandemic phase, both overall and for infectious conditions. It should be noticed, however, that most of these studies suffered from a very short observation period and often failed to properly account for seasonality.

References:

1. Bogossian EG, Taccone FS, Izzi A, Yin N, Garufi A, Hublet S, et al. The Acquisition of Multidrug-Resistant Bacteria in Patients Admitted to COVID-19 Intensive Care Units: A Monocentric Retrospective Case Control Study. Microorganisms. 2020;8(11). Epub 2020/11/25. doi: 10.3390/microorganisms8111821. PubMed PMID: 33227956.

2. Castaldi S, Luconi E, Marano G, Auxilia F, Maraschini A, Bono P, et al. Hospital Acquired Infections in COVID-19 patients in sub intensive care unit. Acta Biomed. 2020;91(3):e2020017. Epub 2020/09/15. doi: 10.23750/abm.v91i3.10376. PubMed PMID: 32921713.

3. Contou D, Claudinon A, Pajot O, Micaëlo M, Longuet Flandre P, Dubert M, et al. Bacterial and viral co-infections in patients with severe SARS-CoV-2 pneumonia admitted to a French ICU. Ann Intensive Care. 2020;10(1):119. Epub 2020/09/08. doi: 10.1186/s13613-020-00736-x. PubMed PMID: 32894364; PubMed Central PMCID: PMCPMC7475952.

4. Garcia-Vidal C, Sanjuan G, Moreno-García E, Puerta-Alcalde P, Garcia-Pouton N, Chumbita M, et al. Incidence of co-infections and superinfections in hospitalised patients with COVID-19: a retrospective cohort study. Clin Microbiol Infect. 2020. Epub 2020/08/04. doi: 10.1016/j.cmi.2020.07.041. PubMed PMID: 32745596.

5. Gomez-Simmonds A, Annavajhala MK, McConville TH, Dietz DE, Shoucri SM, Laracy JC, et al. Carbapenemase-producing Enterobacterales causing secondary infections during the COVID-19 crisis at a New York City hospital. The Journal of antimicrobial chemotherapy. 2020. Epub 2020/11/18. doi: 10.1093/jac/dkaa466. PubMed PMID: 33202023.

6. Li J, Wang J, Yang Y, Cai P, Cao J, Cai X, et al. Etiology and antimicrobial resistance of secondary bacterial infections in patients hospitalized with COVID-19 in Wuhan, China: a retrospective analysis. Antimicrob Resist Infect Control. 2020;9(1):153. Epub 2020/09/24. doi: 10.1186/s13756-020-00819-1. PubMed PMID: 32962731.

7. Luyt CE, Sahnoun T, Gautier M, Vidal P, Burrel S, Pineton de Chambrun M, et al. Ventilator-associated pneumonia in patients with SARS-CoV-2-associated acute respiratory distress syndrome requiring ECMO: a retrospective cohort study. Ann Intensive Care. 2020;10(1):158. Epub 2020/11/25. doi: 10.1186/s13613-020-00775-4. PubMed PMID: 33230710.

8. Montrucchio G, Corcione S, Sales G, Curtoni A, De Rosa FG, Brazzi L. Carbapenem resistant Klebsiella pneumoniae in ICU-admitted COVID-19 Patients: Keep an eye on the ball. J Glob Antimicrob Resist. 2020;23:398-400. Epub 2020/11/27. doi: 10.1016/j.jgar.2020.11.004. PubMed PMID: 33242674; PubMed Central PMCID: PMCPMC7682477.

9. Nori P, Cowman K, Chen V, Bartash R, Szymczak W, Madaline T, et al. Bacterial and fungal coinfections in COVID-19 patients hospitalized during the New York City pandemic surge. Infect Control Hosp Epidemiol. 2020:1-5. Epub 2020/07/25. doi: 10.1017/ice.2020.368. PubMed PMID: 32703320; PubMed Central PMCID: PMCPMC7417979.

10. Para O, Caruso L, Ronchetti M, Finocchi M, Guidi S, Spinicci M. Superinfection with difficult-to-treat bacteria in COVID-19 patients: a call for compliance with diagnostic and antimicrobial stewardship. Intern Emerg Med. 2020:1-3. Epub 2020/11/23. doi: 10.1007/s11739-020-02537-3. PubMed PMID: 33222117; PubMed Central PMCID: PMCPMC7680555.

11. Tiri B, Sensi E, Marsiliani V, Cantarini M, Priante G, Vernelli C, et al. Antimicrobial Stewardship Program, COVID-19, and Infection Control: Spread of Carbapenem-Resistant Klebsiella Pneumoniae Colonization in ICU COVID-19 Patients. What Did Not Work? J Clin Med. 2020;9(9). Epub 2020/08/29. doi: 10.3390/jcm9092744. PubMed PMID: 32854334.

12. Yu D, Ininbergs K, Hedman K, Giske CG, Strålin K, Özenci V. Low prevalence of bloodstream infection and high blood culture contamination rates in patients with COVID-19. PLoS One. 2020;15(11):e0242533. Epub 2020/11/24. doi: 10.1371/journal.pone.0242533. PubMed PMID: 33226995.

13. Goncalves Mendes Neto A, Lo KB, Wattoo A, Salacup G, Pelayo J, DeJoy R, 3rd, et al. Bacterial Infections and Patterns of Antibiotic Use in Patients with COVID-19. J Med Virol. 2020. Epub 2020/08/19. doi: 10.1002/jmv.26441. PubMed PMID: 32808695.
14. Karami Z, Knoop BT, Dofferhoff ASM, Blaauw MJT, Janssen NA, van Apeldoorn M, et al. Few bacterial co-infections but frequent empiric antibiotic use in the early phase of hospitalized patients with COVID-19: results from a multicentre retrospective cohort study in The Netherlands. Infect Dis (Lond). 2020:1-9. Epub 2020/10/27. doi: 10.1080/23744235.2020.1839672. PubMed PMID: 33103530.
15. Liu H, Gao J, Wang Y, Jie J, Luo J, Xu Y, et al. Epidemiological and clinical characteristics of 2019 novel coronavirus disease (COVID-19) in Jilin, China: A descriptive study. Medicine (Baltimore). 2020;99(47):e23407. Epub 2020/11/22. doi: 10.1097/md.0000000000023407. PubMed PMID: 33217886.
16. Rothe K, Feihl S, Schneider J, Wallnöfer F, Wurst M, Lukas M, et al. Rates of bacterial co-infections and antimicrobial use in COVID-19 patients: a retrospective cohort study in light of antibiotic stewardship. Eur J Clin Microbiol Infect Dis. 2020. Epub 2020/11/04. doi: 10.1007/s10096-020-04063-8. PubMed PMID: 33140176.
17. Seaton RA, Gibbons CL, Cooper L, Malcolm W, McKinney R, Dundas S, et al. Survey of antibiotic and antifungal prescribing in patients with suspected and confirmed COVID-19 in Scottish hospitals. J Infect. 2020. Epub 2020/09/29. doi: 10.1016/j.jinf.2020.09.024. PubMed PMID: 32987097; PubMed Central PMCID: PMCPMC7518971.
18. Shin DH, Kang M, Song KH, Jung J, Kim ES, Kim HB. A Call for Antimicrobial Stewardship in Patients with COVID-19: A Nationwide Cohort Study in Korea. Clin Microbiol Infect. 2020. Epub 2020/11/03. doi: 10.1016/j.cmi.2020.10.024. PubMed PMID: 33137513; PubMed Central PMCID: PMCPMC7604125.
19. Stevens RW, Jensen K, O'Horo JC, Shah A. Antimicrobial prescribing practices at a tertiary-care center in patients diagnosed with COVID-19 across the continuum of care. Infect Control Hosp Epidemiol. 2020:1-4. Epub 2020/07/25. doi: 10.1017/ice.2020.370. PubMed PMID: 32703323; PubMed Central PMCID: PMCPMC7417978 authors report no conflicts of interest relevant to this article.
20. Townsend L, Hughes G, Kerr C, Kelly M, O'Connor R, Sweeney E, et al. Bacterial pneumonia coinfection and antimicrobial therapy duration in SARS-CoV-2 (COVID-19) infection. JAC Antimicrob Resist. 2020;2(3):dlaa071. Epub 2020/08/31. doi: 10.1093/jacamr/dlaa071. PubMed PMID: 32864608; PubMed Central PMCID: PMCPMC7446659.
21. Vaughn VM, Gandhi T, Petty LA, Patel PK, Prescott HC, Malani AN, et al. Empiric Antibacterial Therapy and Community-onset Bacterial Co-infection in Patients Hospitalized with COVID-19: A Multi-Hospital Cohort Study. Clin Infect Dis. 2020. Epub 2020/08/22. doi: 10.1093/cid/ciaa1239. PubMed PMID: 32820807.
22. Wang L, Amin AK, Khanna P, Aali A, McGregor A, Bassett P, et al. An observational cohort study of bacterial co-infection and implications for empirical antibiotic therapy in patients presenting with COVID-19 to hospitals in North West London. The Journal of antimicrobial chemotherapy. 2020. Epub 2020/11/14. doi: 10.1093/jac/dkaa475. PubMed PMID: 33185241.
23. Abena PM, Decloedt EH, Bottieau E, Suleman F, Adejumo P, Sam-Agudu NA, et al. Chloroquine and Hydroxychloroquine for the Prevention or Treatment of COVID-19 in Africa: Caution for Inappropriate Off-label Use in Healthcare Settings. Am J Trop Med Hyg. 2020;102(6):1184-8. Epub 2020/04/24. doi: 10.4269/ajtmh.20-0290. PubMed PMID: 32323646; PubMed Central PMCID: PMCPMC7253100.

24. Arshad M, Mahmood SF, Khan M, Hasan R. Covid -19, misinformation, and antimicrobial resistance. Bmj. 2020;371:m4501. Epub 2020/11/26. doi: 10.1136/bmj.m4501. PubMed PMID: 33234500.
25. Comber SDW, Upton M, Lewin S, Powell N, Hutchinson TH. COVID-19, antibiotics and One Health: a UK environmental risk assessment. The Journal of antimicrobial chemotherapy. 2020. Epub 2020/08/14. doi: 10.1093/jac/dkaa338. PubMed PMID: 32785691.
26. DeJong C, Wachter RM. The Risks of Prescribing Hydroxychloroquine for Treatment of COVID-19-First, Do No Harm. JAMA internal medicine. 2020;180(8):1118-9. Epub 2020/04/30. doi: 10.1001/jamainternmed.2020.1853. PubMed PMID: 32347894.
27. Getahun H, Smith I, Trivedi K, Paulin S, Balkhy HH. Tackling antimicrobial resistance in the COVID-19 pandemic. Bull World Health Organ. 2020;98(7):442-a. Epub 2020/08/04. doi: 10.2471/blt.20.268573. PubMed PMID: 32742026; PubMed Central PMCID: PMCPMC7375214.
28. Ginsburg AS, Klugman KP. COVID-19 pneumonia and the appropriate use of antibiotics. Lancet Glob Health. 2020. Epub 2020/11/15. doi: 10.1016/s2214-109x(20)30444-7. PubMed PMID: 33188730.
29. Huttner BD, Catho G, Pano-Pardo JR, Pulcini C, Schouten J. COVID-19: don't neglect antimicrobial stewardship principles! Clin Microbiol Infect. 2020;26(7):808-10. Epub 2020/04/30. doi: 10.1016/j.cmi.2020.04.024. PubMed PMID: 32360446.
30. Miranda C, Silva V, Capita R, Alonso-Calleja C, Igrejas G, Poeta P. Implications of antibiotics use during the COVID-19 pandemic: present and future. The Journal of antimicrobial chemotherapy. 2020. Epub 2020/08/25. doi: 10.1093/jac/dkaa350. PubMed PMID: 32830266.
31. Monnet DL, Harbarth S. Will coronavirus disease (COVID-19) have an impact on antimicrobial resistance? Euro Surveill. 2020;25(45). Epub 2020/11/14. doi: 10.2807/1560-7917.Es.2020.25.45.2001886. PubMed PMID: 33183403.
32. Nieuwlaat R, Mbuagbaw L, Mertz D, Burrows L, Bowdish DME, Moja L, et al. COVID-19 and Antimicrobial Resistance: Parallel and Interacting Health Emergencies. Clin Infect Dis. 2020. Epub 2020/06/17. doi: 10.1093/cid/ciaa773. PubMed PMID: 32544232; PubMed Central PMCID: PMCPMC7337675.
33. Pulia MS, Wolf I, Schulz LT, Pop-Vicas A, Schwei RJ, Lindenauer PK. COVID-19: An Emerging Threat to Antibiotic Stewardship in the Emergency Department. West J Emerg Med. 2020;21(5):1283-6. Epub 2020/09/25. doi: 10.5811/westjem.2020.7.48848. PubMed PMID: 32970587.
34. Rawson TM, Ming D, Ahmad R, Moore LSP, Holmes AH. Antimicrobial use, drug-resistant infections and COVID-19. Nat Rev Microbiol. 2020;18(8):409-10. Epub 2020/06/04. doi: 10.1038/s41579-020-0395-y. PubMed PMID: 32488173; PubMed Central PMCID: PMCPMC7264971.
35. Rawson TM, Moore LSP, Castro-Sanchez E, Charani E, Davies F, Satta G, et al. COVID-19 and the potential long-term impact on antimicrobial resistance. J Antimicrob Chemother. 2020;75(7):1681-4. Epub 2020/05/21. doi: 10.1093/jac/dkaa194. PubMed PMID: 32433765; PubMed Central PMCID: PMCPMC7314000.
36. Vaillancourt M, Jorth P. The Unrecognized Threat of Secondary Bacterial Infections with COVID-19. mBio. 2020;11(4). Epub 2020/08/10. doi: 10.1128/mBio.01806-20. PubMed PMID: 32769090.
37. Youngs J, Wyncoll D, Hopkins P, Arnold A, Ball J, Bicanic T. Improving antibiotic stewardship in COVID-19: Bacterial co-infection is less common than with influenza. J Infect. 2020;81(3):e55-e7. Epub 2020/07/01. doi: 10.1016/j.jinf.2020.06.056. PubMed PMID: 32593654; PubMed Central PMCID: PMCPMC7316044.

38. Clancy CJ, Nguyen MH. COVID-19, superinfections and antimicrobial development: What can we expect? Clin Infect Dis. 2020. Epub 2020/05/04. doi: 10.1093/cid/ciaa524. PubMed PMID: 32361747; PubMed Central PMCID: PMCPMC7197597.
39. Fattorini L, Creti R, Palma C, Pantosti A. Bacterial coinfections in COVID-19: an underestimated adversary. Ann Ist Super Sanita. 2020;56(3):359-64. Epub 2020/09/23. doi: 10.4415/ann_20_03_14. PubMed PMID: 32959802.
40. Kotwani A, Gandra S. Potential pharmacological agents for COVID-19. Indian J Public Health. 2020;64(Supplement):S112-s6. Epub 2020/06/05. doi: 10.4103/ijph.IJPH_456_20. PubMed PMID: 32496239.
41. Langford BJ, So M, Raybardhan S, Leung V, Westwood D, MacFadden DR, et al. Bacterial co-infection and secondary infection in patients with COVID-19: a living rapid review and meta-analysis. Clin Microbiol Infect. 2020. Epub 2020/07/28. doi: 10.1016/j.cmi.2020.07.016. PubMed PMID: 32711058.
42. Lansbury L, Lim B, Baskaran V, Lim WS. Co-infections in people with COVID-19: a systematic review and meta-analysis. J Infect. 2020;81(2):266-75. Epub 2020/05/31. doi: 10.1016/j.jinf.2020.05.046. PubMed PMID: 32473235; PubMed Central PMCID: PMCPMC7255350.
43. Mirzaei R, Goodarzi P, Asadi M, Soltani A, Aljanabi HAA, Jeda AS, et al. Bacterial co-infections with SARS-CoV-2. IUBMB Life. 2020. Epub 2020/08/10. doi: 10.1002/iub.2356. PubMed PMID: 32770825.
44. Rawson TM, Moore LSP, Zhu N, Ranganathan N, Skolimowska K, Gilchrist M, et al. Bacterial and fungal co-infection in individuals with coronavirus: A rapid review to support COVID-19 antimicrobial prescribing. Clin Infect Dis. 2020. Epub 2020/05/03. doi: 10.1093/cid/ciaa530. PubMed PMID: 32358954; PubMed Central PMCID: PMCPMC7197596.
45. Abelenda-Alonso G, Padullés A, Rombauts A, Gudiol C, Pujol M, Alvarez-Pouso C, et al. Antibiotic prescription during the COVID-19 pandemic: A biphasic pattern. Infect Control Hosp Epidemiol. 2020;41(11):1371-2. Epub 2020/07/31. doi: 10.1017/ice.2020.381. PubMed PMID: 32729437; PubMed Central PMCID: PMCPMC7426604.
46. Buehrle DJ, Decker BK, Wagener MM, Adalja A, Singh N, McEllistrem MC, et al. Antibiotic Consumption and Stewardship at a Hospital outside of an Early Coronavirus Disease 2019 Epicenter. Antimicrob Agents Chemother. 2020;64(11). Epub 2020/08/21. doi: 10.1128/aac.01011-20. PubMed PMID: 32816693; PubMed Central PMCID: PMCPMC7577150.
47. Dieringer TD, Furukawa D, Graber CJ, Stevens VW, Jones MM, Rubin M, et al. Inpatient antibiotic utilization in the Veterans Administration during the COVID-19 pandemic. Infect Control Hosp Epidemiol. 2020:1-9. Epub 2020/10/21. doi: 10.1017/ice.2020.1277. PubMed PMID: 33077000.
48. Nestler M, Godbout E, Lee K, Kim J, Noda AJ, Taylor P, et al. Impact of COVID-19 on Pneumonia-Focused Antibiotic Use at an Academic Medical Center. Infect Control Hosp Epidemiol. 2020:1-9. Epub 2020/07/24. doi: 10.1017/ice.2020.362. PubMed PMID: 32698920.
49. Staub MB, Beaulieu RM, Graves J, Nelson GE. Changes in Antimicrobial Utilization During the COVID-19 Pandemic after Implementation of a Multispecialty Clinical Guidance Team. Infect Control Hosp Epidemiol. 2020:1-28. Epub 2020/10/27. doi: 10.1017/ice.2020.1291. PubMed PMID: 33100250.
50. Velasco-Arnaiz E, López-Ramos MG, Simó-Nebot S, Jordan I, Ríos-Barnés M, Urrea-Ayala M, et al. Pediatric antimicrobial stewardship in the COVID-19 outbreak. Infect Control Hosp Epidemiol. 2020:1-3. Epub 2020/06/25. doi: 10.1017/ice.2020.312. PubMed PMID: 32576298; PubMed Central PMCID: PMCPMC7338437.
51. Gonzalez-Zorn B. Antibiotic use in the COVID-19 crisis in Spain. Clin Microbiol Infect. 2020. Epub 2020/11/30. doi: 10.1016/j.cmi.2020.09.055. PubMed PMID: 33248926; PubMed Central PMCID: PMCPMC7688281.

52. Katz SE, Spencer H, Zhang M, Banerjee R. Impact of the COVID-19 Pandemic on Infectious Diagnoses and Antibiotic Use in Pediatric Ambulatory Practices. Journal of the Pediatric Infectious Diseases Society. 2020. doi: 10.1093/jpids/piaa124.
