# Supplementary material for: Sales of antibiotics and hydroxychloroquine in India during the COVID-19 epidemic: An interrupted time series analysis
Source: PLoS Med. 2021 Jul 1;18(7):e1003682. doi: 10.1371/journal.pmed.1003682 (PMC8248656; doi:10.1371/journal.pmed.1003682)
Supplement: S2 Text — (PDF) [file pmed.1003682.s017.pdf]

S2 Text: Detailed methods

1. Interrupted time series analysis

We conducted segmented regression analyses of time series data to assess how much the spread of SARS-CoV-2 infection affected monthly sales volumes of the following drug groups, excluding child-appropriate formulations (CAF): 1) total antibiotics (including azithromycin), 2) azithromycin alone, and 3) hydroxychloroquine (HCQ) [1-5].

1.1 Robustness of sequential measures

Both pre-epidemic and epidemic-time data points were collected at regular monthly intervals, and no major concerns exist with respect to possible changes in the collection method over the study period. A total of 27 data points before the start of the epidemic were available for our analyses. On the one hand, this might not be enough to properly identify underlying seasonal trends. On the other hand, demographic, environmental and epidemiologic characteristics can be considered reasonably stable throughout the relatively short study period (3 years), the only exception being the spread of SARS-CoV-2 infection with its direct and indirect effects. Therefore, underlying historical trends were considered to be minimal and very unlikely to affect our estimates [2]. The effects of the ban on irrational fixed-dose combinations (FDCs) issued in September 2018, likely observable from 2019 onwards, were expected to be more qualitative (type of antibiotics being prescribed and sold) rather than quantitative, thus producing minor changes to the overall volume of antibiotic sales. Owing to the very limited number of data points available before this policy change, we could not adequately evaluate the potential changes it produced as compared to the previous period, but this is very unlikely to affect our estimates.

1.2 Model specification and checking

1.2.1 Impact of COVID-19 on non-CAF antibiotic sales

First, we used general linear models with least-squares estimation to predict the effect of the epidemic on sales volumes, without correcting for seasonality:

Model 1: $Y_m = \beta_0 + \beta_1 T + \beta_2 X + \beta_3 X_{up} T + \beta_4 X_{down} T + \varepsilon$

where:

- Y_m is the outcome, representing monthly sales volume expressed in Standard Units (SU);
- T is the time unit, expressed in months (from January 2018 to September 2020);
- X is a dummy variable representing the pre-epidemic period (up to March 2020, X = 0) and the epidemic period (from April 2020 onwards, X = 1);
- $X_{up}T$ is a scaled interaction term with time that takes value 0 before the epidemic and until the end of the lockdown period (i.e. May 2020 inclusive), and subsequently increases by one-unit each month;
- $X_{down}T$ is a scaled interaction term with time that takes value 0 until the peak of the epidemic wave (i.e. September 2020), and subsequently increases by one-unit each month;
- β_0 is the intercept, interpreted as the outcome value at the beginning of the observation period (January 2018);
- β_1 is the pre-epidemic trend;
- β_2 is the average change in level for the initial phase of the epidemic (i.e. during the preventive lockdown period);
- β_3 is the slope or trend change in the outcome after the lockdown phase;
- β_4 is the slope or trend change in the outcome after the epidemic peak relative to the rising phase of the epidemic wave;

- ε is the error term.

However, Model 1 above did not account for autocorrelation, thus failing to accurately estimate both level and slope changes due to the epidemic [1, 2, 6].
The outcome (sales volume of all antibiotics) was found to follow a fairly normal distribution, but the visual inspection of correlograms suggested the presence of autocorrelation likely attributable to seasonality. Partial autocorrelation (i.e. correlation between non-consecutive values) was also observed, and a similar pattern could be identified in the distribution of residuals after fitting Model 1 without seasonal correction (**S1 Fig**).

In order to account for seasonality, we modified Model 1 and included a fixed effect term *Z* for the rainy season (July to October) [5, 7], during which antimicrobial use seems to peak substantially as compared to the rest of the year:

Model 2: $Y_m = \beta_0 + \beta_1 T + \beta_2 X + \beta_3 X_{up} T + \beta_4 X_{down} T + \beta_5 Z + \varepsilon$

where β_5 represents the average change in level of Y for the rainy months compared to the other months.

The inclusion of a single dummy variable for the rainy season rather than several dummies for each month of the year or for quarters allowed to properly correct for seasonality without incurring in over-parameterization [7, 8].
The distribution of residuals improved substantially as compared to the previous model. Furthermore, the Durbin-Watson test statistic was not indicative of autocorrelation (DW = 2.0449), and the Ljung-Box test at lag 12 showed no evidence of lack of fit (P = 0.5077) [6]. We therefore chose Model 2 to estimate the impact of COVID-19 epidemic on monthly sales volume of antibiotics.

1.2.2 Impact of COVID-19 on azithromycin sales

In order to examine the effect of the epidemic on azithromycin sales volumes, we followed the same steps described above for antibiotic sales. The outcome variable was found to be stationary, similarly to what observed with the previous one. However, in the case of azithromycin sales, Model 2 (segmented regression with a fixed effect term for the rainy season) failed to properly account for the underlying seasonal and non-seasonal trends. For this reason, we opted for an alternative approach to seasonality adjustment, replacing the fixed effect term for rainy season with sine and cosine functions of time (harmonic seasonal model or Fourier terms) [5, 7, 9]. Only those found to be statistically significant (P < 0.05) were retained in the model. Yet, we still found evidence of residual serial correlation and further modified the model by including autocorrelated errors up to lag 6 as follows:

Model 3: $Y_m = \beta_0 + \beta_1 T + \beta_2 X + \beta_3 X_{up} T + \beta_4 X_{down} T + sin1 + cos1 + sin2 + cos2 + R_m$

where *sin1*, *cos1*, *sin2* and *cos2* are the Fourier terms and R_m is the error term inclusive of autocorrelated errors ($R_m = \phi_1 + \phi_2 + \phi_3 + \phi_4 + \phi_5 + \phi_6 + \varepsilon$).
The model's goodness-of-fit substantially improved, as suggested by the Durbin-Watson test (DW = 1.8651), the Ljung-Box test at lag 12 (P = 0.57), the distribution of residuals and the visual inspection of the autocorrelation and partial autocorrelation function (**S2 Fig**). Model 3 was therefore selected to assess the impact of the epidemic on azithromycin sales volumes.

1.2.3 Impact of COVID-19 on HCQ sales

Contrary to the two outcomes examined previously, the distribution of monthly sales volume of HCQ was clearly skewed, particularly during the epidemic period. Because the stationary assumption was not satisfied and we lacked sufficient data points to use ARIMA models, we performed an exploratory analysis as detailed below. We first fitted Model 1 using an alternative definition of X and XT, such that COVID-19 epidemic was set to start in March 2020 and the effect of lockdown was ignored. Our choice was motivated by the visual inspection of crude time trends of HCQ sales showing an unexpected peak in March 2020, along with multiple reports from the field indicating a massive use of HCQ especially in prophylactic regimens. Hence, we fitted our model as follows:

Model 4: $Y_m = \beta_0 + \beta_1 T + \beta_2 W + \beta_3 W_{up} T + \beta_4 W_{down} T + \varepsilon$

where:

- Y_m is the outcome, representing monthly sales volume expressed in Standard Units (SU);
- T is the time unit, expressed in months (from January 2018 to September 2020);
- W is a dummy variable representing the pre-epidemic period (up to February 2020, W = 0) and the epidemic period (from March 2020 onwards, W = 1);
- $W_{up}T$ is a scaled interaction term with time that takes value 0 before the epidemic (i.e. March 2020 inclusive), and subsequently increases by one-unit each month;
- $W_{down}T$ is a scaled interaction term with time that takes value 0 until the epidemic peak (i.e. September 2020), and subsequently increases by one-unit each month throughout the declining phase of the epidemic wave;
- β_0 is the intercept, interpreted as the outcome value at the beginning of the observation period (January 2018);
- β_1 is the pre-epidemic trend;
- β_2 is the average change in level at the start of the epidemic;
- β_3 is the slope or trend change in the outcome from March 2020 onwards;
- β_4 is the slope or trend change in the outcome from October 2020 relative to the rising phase of the epidemic wave;
- ε is the error term.

Since HCQ is predominantly used as an immunomodulator and most commonly for non-infectious conditions, adjustment for seasonality was deemed unnecessary, and the model did not improve after adding a fixed effect for the rainy season or using alternative approaches for seasonality adjustment as done with total antibiotic sales and azithromycin sales. In order to account for the remaining serial correlation in the data, we corrected the model through the inclusion of an autocorrelated error term for lag 1:

Model 5: $Y_m = \beta_0 + \beta_1 T + \beta_2 W + \beta_3 W_{up} T + \beta_4 W_{down} + R_m$

where $R_m = \phi_1 + \varepsilon$.

The Durbin-Watson statistic (DW = 1.8628) and Ljung-Box test at lag 12 (P = 0.8747) were indicative of an acceptable goodness-of-fit for Model 5, and the autocorrelation function plot was also comforting. Yet, some degree of deviation from normality was observed in the distribution of residuals (**S3 Fig**). These considerations along with the known violation of the stationary assumption impose caution in interpreting the results of this model.

1.3 Anticipated challenges and mitigation strategies

Although interrupted time-series (ITS) analysis was considered as the best approach for this study, this method does have limitations that were carefully evaluated and addressed.

First, the correct specification of the underlying trend and seasonality is key to build the most appropriate model but, as mentioned previously, it is limited by the amount of available data points in the time series. A visual inspection of the series of each outcome seemed to suggest that the underlying trend was fairly linear after accounting for seasonal cycles. Furthermore, a range of approaches were explored to ensure the best possible adjustment for seasonality. In particular, a single fixed effect term for the rainy season was found to perform reasonably well in the model for non-CAF antibiotic sales. Attempts were also made with Fourier terms and spline functions of time, but the model with the fixed effect term resulted to be the best. In contrast, sine and cosine functions of time were successfully included in the model for azithromycin sales as this approach showed a better performance as compared to the alternatives. In addition, to capture non-seasonal trends, further adjustments were necessary to account for the remaining serial correlation in the data. We thus included autocorrelated errors until optimization of the autocorrelation and partial autocorrelation functions. With regards to the model for HCQ sales, autocorrelated errors were also used, but no seasonal adjustment was required given the different pattern of use of this drug.

For the selected models, residuals were found to behave as white noise, suggesting that the models might be correctly specified [10].

Second, the likelihood of co-occurring events that could have acted as confounding factors was considered negligible. As discussed in section 1.1, no significant historical changes were anticipated.

Third, one of the assumptions of ITS analysis is that no major changes occurred in the way outcomes were recorded over the study period. Although the exact data collection method utilized by IQVIA Inc. has never been publicly released, their datasets have been used in several studies so far and are generally considered of very good quality. No significant modifications have been introduced in the collection approach over the last 3 years, thus making us confident that the aforementioned assumption is satisfied.

2. Estimation of excess treatment courses from sales data

The IQVIA dataset available for our study includes dosage information for each formulation. While it is not possible to provide a reasonable and reliable estimate of the excess treatment courses from total antibiotics sales considered as a whole, we did so for azithromycin alone.

From our model, we found that, for the 4-month period from June to September 2020 (i.e. during the rising phase of the epidemic wave), over 99.5% of the formulations were either 500 mg (62%) or 250 mg (38%). According to the Indian national guidelines for antimicrobial use [11], a single treatment course of azithromycin for respiratory tract infections is 500 mg once a day for 5 days.

Hence, we computed the number excess treatment courses as follows:

$$0.62 * \frac{38.0\ million\ doses\ sold}{5\ days} + 0.38 * \frac{\left(\frac{38.0\ million\ doses\ sold}{5\ days}\right)}{2} = 6.2 \text{ million treatment courses.}$$

References

1. Lopez Bernal J, Soumerai S, Gasparrini A. A methodological framework for model selection in interrupted time series studies. J Clin Epidemiol. 2018;103:82-91. Epub 2018/06/10. doi: 10.1016/j.jclinepi.2018.05.026. PubMed PMID: 29885427.

2. Lopez Bernal J, Cummins S, Gasparrini A. Interrupted time series regression for the evaluation of public health interventions: a tutorial. Int J Epidemiol. 2017;46(1):348-55. Epub 2016/06/11. doi: 10.1093/ije/dyw098. PubMed PMID: 27283160; PubMed Central PMCID: PMCPMC5407170.
3. Xiao H, Augusto O, Wagenaar BH. Reflection on modern methods: a common error in the segmented regression parameterization of interrupted time-series analyses. Int J Epidemiol. 2020. Epub 2020/10/25. doi: 10.1093/ije/dyaa148. PubMed PMID: 33097937.
4. Wagner AK, Soumerai SB, Zhang F, Ross-Degnan D. Segmented regression analysis of interrupted time series studies in medication use research. J Clin Pharm Ther. 2002;27(4):299-309. Epub 2002/08/14. doi: 10.1046/j.1365-2710.2002.00430.x. PubMed PMID: 12174032.
5. Shumway RH, Stoffer DS. Time series analysis and its applications : with R examples. 2017.
6. Linden A. Conducting Interrupted Time-series Analysis for Single- and Multiple-group Comparisons. The Stata Journal. 2015;15(2):480-500. doi: 10.1177/1536867X1501500208.
7. Bhaskaran K, Gasparrini A, Hajat S, Smeeth L, Armstrong B. Time series regression studies in environmental epidemiology. Int J Epidemiol. 2013;42(4):1187-95. Epub 2013/06/14. doi: 10.1093/ije/dyt092. PubMed PMID: 23760528; PubMed Central PMCID: PMCPMC3780998.
8. Zhang F, Wagner AK, Soumerai SB, Ross-Degnan D. Methods for estimating confidence intervals in interrupted time series analyses of health interventions. J Clin Epidemiol. 2009;62(2):143-8. Epub 2008/11/18. doi: 10.1016/j.jclinepi.2008.08.007. PubMed PMID: 19010644; PubMed Central PMCID: PMCPMC3828652.
9. Jebb AT, Tay L, Wang W, Huang Q. Time series analysis for psychological research: examining and forecasting change. Frontiers in Psychology. 2015;6(727). doi: 10.3389/fpsyg.2015.00727.
10. Woodward WA, Gray HL, Elliott AC. Model identification. Applied time series analysis with R. 2nd ed: CRC Press; 2017.
11. National Standard Treatment Guidelines for Antimicrobial Use in Infectious Diseases. New Delhi, India: National Centre for Disease Control, Directorate General of Health Services, Ministry of Health & Family Welfare, Government of India, 2016.
