# Supplementary material for: Sales of antibiotics and hydroxychloroquine in India during the COVID-19 epidemic: An interrupted time series analysis
Source: PLoS Med. 2021 Jul 1;18(7):e1003682. doi: 10.1371/journal.pmed.1003682 (PMC8248656; doi:10.1371/journal.pmed.1003682)
Supplement: Attachment — Submitted filename: Response to Reviewers.docx [file pmed.1003682.s018.docx]

**Point by point response to reviewers**

**Reviewer #1**
Thank you for the opportunity to read and comment on your study. You have collected a lot of data and it is interesting to get to understand more about prescribing during the pandemic. My understanding of the aim is that you wanted to assess the impact of COVID-19 pandemic in India on the national consumption of antibiotics before-and-after, and to determine the impact of COVID-19 on antibiotic use in general, azithromycin and hydroxychloroquine.

Methods
The main outcomes of interest for this study was the sales volume of antibiotics and HCQ. The drug sales data was obtained from IQVIA Inc. From your information, we understand that IQVIA Inc. collects over-the-counter (OTC) and prescription-based sales data in India through a representative panel of drug stockists and offers an overall 95% coverage of the total pharmaceutical market combining the retail sector, hospitals and dispensing doctors. I have some questions to the collection of drug data.
1) For me this was a bit blurry - I understand that the sales data then cover hospitals and out-patients, but please explain: if you have a representative sample then the 95% coverage is actually an estimate and not true coverage?

**In India, IQVIA has a panel of approximately 9,500 Stockists whose monthly sales is then projected to the universe of approximately 30,000 Stockists in the country. IQVIA keeps updating the panel and tracking the overall universe of stockists to ensure the panel is representative. IQVIA performs Stockists Sell-Out Audits (SSA) and Hospital Secondary Audits (HSA), as well as the dispensing doctor’s sector as part of the Total Sales Audit (TSA). The combined audited retail, hospital and dispensing doctors’ sectors is estimated to have accounted for 95% of the total pharmaceutical market in terms of value sales. All audits are based on the panel stockist data which is a robust representation for the entire universe of stockists. The audits have been designed in such a way that the panel data is extrapolated to 100% using projection factors. For this reason, it is an estimate and not true coverage. We have included more details on this in the methods (Lines 155-161).**

2) Furthermore, please add whether the coverage is similar in all geographical regions

**Our data refer to India’s private sector and are nationally representative, which means that any differences across geographic areas within the country are accounted for.**

3) IQVIA Inc. collects data from drug stockists - how long lag-time would you expect from the data sold from the stockiest to the actual sale to patients (hospitals, pharmacies and dispensing doctors - and all do probably also have a stock) - do you think that could have an impact on your figures? (maybe  you could touch upon this in discussion?)

**Based on field experience, the lag-time for antibiotics is usually about 7-10 days for hospital pharmacies but can take a little longer for retail pharmacies (up to 10-15 days or 21 days for some generics). However, please note that during the COVID-19 pandemic this lag-time was shortened for hospital and retail pharmacies due to increase in demand for antibiotics. As suggested by the reviewer we discussed this in the limitation section (Lines 374-379).**

4) Another issue that was contradictive: Under "What did the researchers do and find?" you state  that "Using an interrupted time-series (ITS) design, we examined national sales volumes of total antibiotics, azithromycin alone, and hydroxychloroquine (HCQ) in India's private sector from January 2018 to September 2020". I have always understood "national sales volumes" as total sales (i.e. both private and public sector), but as you stated in discussion: "IQVIA data only cover the private healthcare sector thus…." As I read this, IQVIA Inc. data probably only cover the private sector - I suggest that this is made clearer in methods. Maybe the discussion also should be broadened a bit around this issue and whether the lack of information from the public sector matters? (Or would you say that they are similar e.g. with regard to patients drugs used, antibiotics available etc.?
 **Thank you for this comment. IQVIA only captures sales data from the private sector nationally. This is stated in the methods and reiterated in the discussion. We also mention this among the limitations of our study, because it is not possible to draw conclusions on prescribing/dispensing practices in the public sector. However, as we point out in the paper, it should be noted that the private sector is largely predominant in India and thus it accounts for most antibiotic consumption countrywide. This concept has been reinforced further in the manuscript (Lines 127-129 and Lines 350-364). As suggested by the reviewer, we removed the word “national” throughout the manuscript.**

5) With regard to schedule H/H1, please add where azithromycin is classified. Furthermore, it would also be nice to know the prescription status of HCQ and whether that is a drug that could be sold as OTC.
 **Please note that this information is already included in the manuscript: “Issuing further restrictions on azithromycin prescription by moving it from Schedule H to H1 as done with HCQ could potentially help limit the widespread use of this important antibiotic.” We have now included further specifications in this regard in the methods (Lines 189-190) and S4 Table.**

Results
6) In result you find that "Antibiotic sales volumes declined in April and May 2020" and then you have added "likely due to the very limited mobility allowed during the lockdown phase" But could another explanation be because it was less access to antibiotics due to mobility - or because it was less need - i.e. lower infection pressure? And further you say "Moreover, we observed a monthly increase in trend after the lockdown period for non-CAF antibiotics" I suggest to addressed this further in discussion.

**Thank you for your comment. We agree with the reviewer that the decline in antibiotics could be due to a combination of limited mobility, lack of access and reduced number of infections. All these can be considered as direct or indirect effects of the lockdown measures.**

Discussion
7) Regarding "HCQ, the sudden increase in consumption registered in March 2020 could be attributed to prophylaxis for healthcare workers as initially recommended by the Ministry of Health". I agree, increased use was seen in many countries in the first days of the pandemic, however HCQ was also given as treatment to COVID-19 patients.

**Thank you for this comment. Although we agree that HCQ was also being used for treatment, it should be noted that until June 2020 the number of cases was still contained in most parts of India and thus unlikely to explain the huge increase in use of HCQ that was observed in March. For this reason, we believe that the high sales volumes in March are predominately indicative of the widespread use in prophylaxis.**

Under limitations
8) With regard to the coverage of data (see also Q in methods); private versus public sector "First, IQVIA data only cover the private healthcare sector thus potentially underestimating the excess use of antibiotics and HCQ due to COVID-19".  Could it be the other way around - that there is excess use in private sector and not in the public sector?

**Thank you for this comment. However, data from the public sector were not available for our analyses and thus we cannot comment on this issue. This is listed among the limitations of our study, and we deleted the interpretation from public sector (Lines 360-364).**

With regard to inpatient versus outpatient use: "Second, our data could not distinguish between inpatient and outpatient use of antibiotics……". Somehow you could, as you state in results; "Similarly, monthly sales of selected parenteral antibiotics that are typically used in inpatient care such as carbapenems, glycopeptides, third generation cephalosporins and polymyxins, has remained almost stable". Don't this indicate that the changes in antibiotics could be more due to the use in outpatients than the use in hospitals?
 **Thank you for this suggestion. It is likely that antibiotics were used more in outpatient settings; however, antibiotics such as azithromycin or doxycycline as well as HCQ – whose consumption increased significantly – are mainly used as oral formulations even in inpatient settings. Thus, it is difficult to make any conclusive statement.**

With regard to seasonal variations in the outcome, see comments in Text S2
 **Reviewed and addressed.**

With regard to other possible treatments for COVID, you mention that ivermectin is also used off-label for COVID-19 treatment in India, but what about other treatment regimens that have been discussed during the pandemic e.g. corticosteroids, some antivirals etc?
 **We agree with the reviewer that this statement does not account for many other drugs that have been repurposed for use in the management of COVID-19. We have deleted that sentence to avoid potentially misleading conclusions.**

Then you mention DDDs: " Finally, we did not have data in daily defined doses (DDDs); however, the antibiotic consumption data in standard units correlates very well with DDDs"  But your reference (no. 46) refers to DOT and not to DDDs.
 **Thank you for pointing this out. We edited as follows “there was a very good correlation between the DDD and standard units when estimating antibiotic consumption per person” (Lines 372-374).**

Text S2: Detailed methods, model 4
Here you claim that HCQ is predominantly used as an immunomodulator and most commonly for non-infectious conditions, hence adjustment for seasonality was deemed unnecessary". However, HCQ is also indicated for Malaria (both prophylaxis and therapy) - and those are approved indications among others both in Europe and in US (FDA ), therefore it is strange if you do not have those indications in India. Moreover, according to an article about  Seasonal variations in incidence of severe and complicated malaria in central India (Indian J Med Sci. 2001 Jan;55(1):43-6.) claims that the maximum prevalence of malaria in most parts of India is from July to November months, which is the malaria season in India and are among the months you address in the study. It would be nice to address this point in discussion.

**Thank you for this comment. We understand your concerns, but we want to reiterate that HCQ is not recommended for treatment of malaria in India, and we received confirmation from the field that this is not part of common practice. In the methods section of the paper (Lines 217-218), we refer to this point and explicitly state that HCQ is not utilized for malaria treatment. We also added in the introduction section on the general indications of azithromycin and HCQ in India as per reviewer 3 suggestion (Lines 103-106).**

Table S4: List of all antimicrobials included in our dataset, along with AWaRe (2019) and ATC categories (2020).
In the column for ATC you have grouped antibacterials, but not necessarily according to ATC. If you want to name the column ATC you should use the ATC code in addition to the antibiotic group, e.g. J01G Aminoglycosides. I also see that you diverge from the ATC groups for some of the substances e.g. tigecycline is grouped under tetracyclines (J01AA) in the ATC-system. It is of course OK to group the antibiotics the way you decide, and it is very clear in the Table how you group them but be aware that it is not ATC grouping.

**Thanks for pointing this out. The column heading was modified as needed.**

For international readers it would have been nice to have an extra column for which antibiotics are schedule H, H1 and OTC

**All antibiotics are schedule H and none can be dispensed OTC. As suggested by the reviewer, we have added a column in S4 Table to indicate which drugs are categorized as Schedule H or H1.**

Then, in the footnote you have mentioned: "CQ, chloroquine; HCQ, hydroxychloroquine" I could not find that you had used the abbreviations in the Table?
 **Thank you for pointing this out. We have made the necessary corrections.**

**Reviewer #2**
Thanks for the opportunity to review your manuscript. My role is as a statistical reviewer, so my comments and questions are focused on the data and analysis. This manuscript presents an interrupted time-series analysis of national-level sales of antibiotics (AZM and the one we have heard so much about in the last 12 months, HCQ). I've put general comments/queries first and followed this with questions about specific sections of the manuscript.
The manuscript is good quality, it's well-written and clear and the approach to analysis is overall good (the details in the Supplementary appendix weren't just helpful for the review but were interesting to read as well).
The supplementary appendix was a useful addition for my review - the detailed methods you provided were very helpful in my review and answered several questions I had from reading the main manuscript.  The final models selected after observing autocorrelation in the original formulation are appropriate and the limitations with the model for HCQ are acknowledged.
 **Thank you for the positive feedback**

My main concern is about the data used in the analysis. The data is sourced from a private company that estimates sales through sampling over the counter sales and prescription records. This is briefly discussed in S2 Text - that there is a risk that the approach used to estimate national sales becomes biased during the COVID-19 period of 2020. The references (1, 25) provided are for two published studies with international comparisons (which include India) using the same database. I spent some time looking through IQVIA's website, but I didn't find any more detailed information. I was looking for the specific methodology used to estimate the total sales information (including how this was done in India) and any quality assessments of their methodology. The two publications provided to point to this being a legitimate source of data but for the review I would like to see the methodology used to estimate total sales so that I can make a complete and robust assessment of this work. I don't harbor any suspicions here but after the retraction of the work using the Surgisphere dataset last year in The Lancet I would like to be able to fully recommend your work with no doubts about the data used in the analysis.
 **We understand the reviewer’s concern. We want to emphasize that IQVIA is considered as a legitimate data source. In fact, the United States (US) Centers for Disease Control and Prevention recently published the trends in antibiotic use during COVID-19 in the US using the proprietary IQVIA dataset (**[**https://doi.org/10.1093/cid/ciaa1896**](https://doi.org/10.1093/cid/ciaa1896)**)& (https://doi:10.1001/jamainternmed.2020.2594). In addition, the CDC has been using IQVIA dataset to track antibiotic use since 2018 (**[**https://www.cdc.gov/antibiotic-use/stewardship-report/current.html**](https://www.cdc.gov/antibiotic-use/stewardship-report/current.html)**).**

**In India, IQVIA has a panel of approximately 9,500 Stockists whose monthly sales is then projected to the universe of approximately 30,000 Stockists. IQVIA keeps updating the panel and tracking the overall universe of stockists to ensure the panel is representative. IQVIA performs Stockists Sell-Out Audits (SSA) and Hospital Secondary Audits (HSA), as well as the dispensing doctor’s sector as part of the Total Sales Audit (TSA). The combined audited retail, hospital and dispensing doctors’ sectors is estimated to have accounted for 95% of the total pharmaceutical market in terms of value sales. All audits are based on the panel stockist data which is a robust representation for the entire universe of stockists. The audits have been designed in such a way that the panel data is extrapolated to 100% using projection factors. However, the precise details of the projection algorithm are withheld for proprietary reasons.**

**The data collection from stockists is automated and collected electronically. While national level lockdown did bring some challenges, they were largely in terms of delay in receiving the data from stockists for lockdown months but there was no change in methodology.**

P9. The association between sales volume and new COVID-19 cases was analysed with Pearson's correlation. There is a risk with this approach of an inflated correlation coefficient due to common underlying trends captured as an 'association'. There are a variety of approaches to dealing with this e.g. in the environmental epidemiology context I have used GAMs with a non-linear time effect to detrend and get a less biased estimate of association. I would recommend to either use a more appropriate approach (there are several to choose from) or to leave this out and present the summary figures which show a clear pattern of association as is.
 **Thank you for this comment. We agree on leaving this out of the analysis and have modified the paper accordingly.**

In the figures (particularly Fig 1) the x-axis is quite noisy with the month and year displayed for each data point - this could be refined (e.g. year not displayed in each label, not every month labelled on the axis) to be clearer.
 **Thank you for this suggestion. We have revised the figures in order to make them more easily readable.**

In Figure 2, I was unclear about the rate. Is this the rate of new infections per 100,00 in each month, or a cumulative rate of all COVID19 infections to the end of each months?
 **Thank you for this comment. It is cumulative rate of new cases reported per month, obtained by summing up the number of new daily cases. We have added clarifying information in the figure caption and legend.**

Text S2. The method used is described as 'generalized linear models with least-squares', I think this would be more accurately called ''general linear model' if least squares where used with a normal distribution.
 **Corrected as per your suggestion.**

**Reviewer #3:**

This is important data to show the wider impact of COVID-19 on antibiotic use. I have some clarification comments and suggestions for strengthening the introduction and discussion. For example, I think more is needed on the background on azithromycin and HCQ use, as well as what a "lockdown" was in India. There is also some subtlety that could be included around the discussion about when and why antibiotic use might change with COVID-19 (e.g. direct and indirect effects). I cannot assess the time series analysis in detail but I think more justification of the choices made is needed.
 **Thank you for your comment. As per reviewer suggestion, we have added some more background information regarding azithromycin and HCQ use prior to and during the COVID-19 pandemic (Lines 99-106). We have also included some details regarding the definition and implications of the national lockdown in India (Lines 203-207). A detailed description of the models and the justification for our analytical choices is provided in S2 Text.**

Minor comments
Introduction
-       COVID-19 is the disease with symptoms not "infection designated as coronavirus disease". Unclear please change

**Thank you for your comment. We corrected as suggested (Lines 94-95).**

-       Why were people using azithromycin and HCQ? More information on the early evidence on this regimen and subsequent rebuttal

**We have added more background information as suggested (Lines 99-112).**

-       More subtlety is needed around why a patient might receive an antibiotic: the symptoms of hospitalised COVID-19 look like a bacterial pneumonia. Similarly, in the community, it is unlikely that people are taking an antibiotic because they think they have a bacterial infection but more because there are no treatments for COVID-19 and they want to take something

**We understand the reviewer’s concerns. However, the guidelines from WHO as well as those from the Indian Ministry of Health clearly recommend against the use of antibiotics for mild/moderate infections, that represent that vast majority of COVID-19 cases.**

-       What are azithromycin / HCQ used for in India "normally"? (i.e. pre-COVID-19?)
 **Azithromycin is utilized to treat a range of conditions, including acute respiratory tract infections, bacterial dysentery and enteric fever. We have added information in this regard and cited the most recent national treatment guidelines (2019). HCQ is mainly utilized for rheumatological conditions and for post-infection arthritis/arthralgias such as those following Chikungunya infection; of note, this drug is not used for malaria as per Indian guidelines. We added this information in the introduction section as suggested (Lines 99-112).**

Methods
-       Why is the information on schedule H / H1 in there?

**Schedule H and H1 identify drugs that are subject to prescription as per India’s legislation. We believe it is important to underline that all antibiotics are categorized as Schedule H or H1, although over the counter dispensing remains a common practice throughout the country. As suggested by another reviewer, we have included additional information in this regard, which could be helpful for an international reader to familiarize with India-specific regulations concerning antibiotic prescribing.**

-       Why does social distancing / school closures affect why CAF formulas were not included? Suggest to cut this sentence (middle page 9), unless they could also assess the impact of such NPIs on the number of respiratory infections and hence the reduction in CAF antibiotic use.

**There is good evidence from high income countries indicating decreased antibiotic use in children during the pandemic. We believe that the situation in India is not different. We cited references in the original draft and clearly indicated the rationale for not including CAF in the time-series analysis.**

-       The recommendation for HCQ-based prophylaxis should be included in the introduction - was this just for India?

**As suggested by the reviewer, we have added more background information regarding the use of HCQ in treatment and prophylaxis (Lines 99-112).**

-       (Top of page 10) If HCQ is not recommended for malaria why is it a concern to be used more for COVID-19? More background on this needed in the introduction.

**Thank you for this comment. A number of safety concerns have been raised regarding the widespread of HCQ, which is even more compelling in the absence of clinical benefit. We have integrated the discussion with comments on this point (Lines 357-359).**

-       What does it mean " this approach did not perform equally well for azithromycin"? I think the

model needs to be the same for all or at least all the same model types tested for each data set.
 **Predictors used in the model are the same for total antibiotics and azithromycin; we simply utilized different approaches to adjust for seasonal trends. We could have used Fourier terms in the model for antibiotics, but we opted for the most parsimonious model given that a single fixed effect term showed to perform reasonably well. Please see S2 Text for a detailed explanation of our choices.**

Results
-       How are the confidence intervals generated around the proportion of non-CAF? Why do you have confidence intervals? Is it not just a single proportion?

**This is standard practice to provide confidence intervals for proportions. Of note, this helps in comparing percent changes over time (e.g. 2020 vs 2019) in a more appropriate way than, say, p-values.**

-       How did AWaRE classifications vary in the COVID period?

**As stated in the results section of our manuscript, the distribution of AWaRe categories did not change much in 2020 as compared to the previous years. Of note, the median percentage of each AWaRe category remained stable over time, including during the rising phase of the epidemic wave.**

-       Is azithromycin a macrolide? Can you explicitly say that this could have caused the jump? (bottom of page 11).

**We clarified this as suggested by the reviewer (Line 101).**

-       Figure 2: the trend is not completely obvious to me and why is there no trend with HCQ? Can you discuss /explain the non-trends in this graph?

**Please note that Figure 2 shows the crude trends in sales without accounting for any other factor that can explain changes over time. In the specific case of HCQ, it should be noted that even segmented regression analyses did not identify any significant change in trend but only a substantial level change. However, we refer the reviewer to the dedicated section of the manuscript and particularly to Fig 4 for a clear representation of this phenomenon.**

-       Figure 3: the correlation / trend is clearer here: did you analyse this statistically?
 **Figure 3 shows the results of segmented regression analyses as indicated in the caption and notes and extensively described in the methods and S2 Text.**

-       It's not completely clear to my why lockdown would affect antibiotic usage - can explain what "lockdown" meant in the Indian setting and this link in the background.

**Thank you for this comment. As suggested by the reviewer, we have included a more detailed description of the national lockdown enforced in India between April and May 2020, along with clarifications regarding the hypothesized negative effect produced on antibiotic sales (Lines 203-207).**

-       How did doxycycline levels compare to September 2018? Why is this and faropenem singled out for analysis?

**Doxycycline sales volumes in September 2018 were very close to the levels observed in the same month of 2019, which is why we did not explicitly mention this. Besides azithromycin, two other antibiotics (doxycycline and faropenem), which are commonly used for respiratory tract infections, showed increased consumption. Thus, we presented the data on these two antibiotics (Lines 253-254) (Lines 346-347).**

-       Why was there a change in September 2020?

**The epidemic wave in India reached its peak in September 2020. We have now clarified this point in the manuscript (Lines 183-184).**

-       In April 2020 non-CAF and azithromycin decreased vs 2018/19 or the start of 2020?
 **Please note that we did not conduct a before-and-after study but an interrupted time series analysis, which is a much more sophisticated analytical approach for this kind of impact evaluation and allows to estimate changes in level and trend based on previous observations and also accounting for potential confounding due to seasonality etc. Our models estimated a decline in sales in April as compared to pre-epidemic phase (i.e. the previous month), but this also accounts for the baseline level for that particular month as observed in the previous years. We have clarified this throughout the manuscript and its supplementary information.**

Discussion
-       Is azithromycin really often prescribe for this condition? In India private / OTC settings?

**Azithromycin is very commonly prescribed and also dispensed over the counter for acute respiratory symptoms. As suggested by the reviewer, we added details on indications of azithromycin in India in the introduction (Lines 99-112). During the epidemic, this practice has further increased because of the hypothetical benefits against COVID-19, as indicated in our paper. Given that the private sector accounts for 90% of antibiotic sales in India, azithromycin use in this specific sector represents a serious concern for all the reasons we have outlined in the discussion.**

-       Is there a potential that other diseases, not being treated in hospital settings, could explain the increase in some antibiotic usage?

**Please note that our models estimate the excess sales attributable to the epidemic, i.e. an amount that adds to the baseline volume and thus accounts for use for any conditions. Furthermore, health-seeking behaviors have changed during the pandemic as suggested by evidence from high-income countries (**[**https://doi.org/10.3399/BJGP.2020.1051**](https://doi.org/10.3399/BJGP.2020.1051)**), leading many people to abstain from consulting healthcare providers unless very sick and/or unless showing symptoms of acute respiratory infections that could be COVID-19. To our knowledge, there is no evidence of co-occurring epidemics or other unexpected events that could have explained the excess sales in azithromycin and other drugs likely attributable to the ongoing epidemic. Seasonal infections such as dengue, malaria, chikungunya for which antibiotics are used inappropriately also were less reported during year 2020.**

-       In line with the above why might you expect the number for COVID-19 to be an underestimate? Surely if not attending healthcare facilities they may self-medicate?

**COVID-19 cases are underestimated in all countries, and it is well known that this was the case in India as well due to the limited testing capacity particularly in the earlier phases. Yet, a number of individuals with mild symptoms are likely to be given medications, such as azithromycin, either in healthcare facilities or over the counter in pharmacies/drug shops. Because our outcome data are pharmaceutical sales volumes, we are able to capture both prescription-based and over the counter sales (and thus self-medication as well).**

-       Could some of this background on azithromycin use go into the introduction to justify the focus and concern?
**We have added more details on azithromycin use in India in the introduction section (Lines 99-112); however, we believe the concerns are more appropriate in the discussion section.**

-       What proportion of the healthcare prescribing in India is not in the private sector? (to assess the limitations)

**75% of the healthcare is delivered by private providers. We have included this information in the manuscript and reinforced this concept in the discussion (Lines 127-129 and Lines 350-364).**
