# Supplementary material for: Sales of antibiotics and hydroxychloroquine in India during the COVID-19 epidemic: An interrupted time series analysis
Source: PLoS Med. 2021 Jul 1;18(7):e1003682. doi: 10.1371/journal.pmed.1003682 (PMC8248656; doi:10.1371/journal.pmed.1003682)
Supplement: Attachment — Submitted filename: Response to editors & reviewers.docx [file pmed.1003682.s019.docx]

**Requests from Editors:**

Please adapt the title to "Sales of antibiotics and hydroxychloroquine in India during the COVID-19 epidemic: An interrupted time-series analysis" or similar.

**Response: Made changes as suggested**

At line 32, please make that "the private sector".

**Response: Made changes as suggested**

At line 47, would "... first epidemic wave ..." be appropriate?
**Response: Made changes as suggested**

In the abstract and throughout the paper, please quote p values alongside 95% CI where available.
**Response: Made changes as suggested**

Please add a new final sentence to the "Methods and findings" subsection of your abstract, which should begin "Study limitations include ..." or similar and should quote 2-3 of the main study limitations.
**Response: Made changes as suggested. Following statements are included in Abstract “Study limitations include the lack of coverage of the public healthcare sector, the inability to distinguish antibiotic and HCQ sales in inpatient versus outpatient care, and the suboptimal number of pre- and post-epidemic data points that could have prevented an accurate adjustment for seasonal trends despite the robustness of our statistical approaches.”**

At line 82, please make that "... likely to have occurred in other LMICs ...".

**Response: Made changes as suggested**

Early in your Methods section, please state whether or not the study had a protocol or prespecified analysis plan, and if so attach the relevant document(s) as a supplementary file, referred to in the text.
**Response: Made changes as suggested. Following statement is included in Methods section: “Although a formal detailed protocol was not developed, our analytic plan was designed a priori before performing the analyses. However, our original study only included sales data up to September 2020 and was updated during the review process as more recent data became available to the study team.”**

Please highlight non-prespecified analyses.
**Response: Made changes as suggested. Following statement is included in Methods section: “Although a formal detailed protocol was not developed, our analytic plan was designed a priori before performing the analyses. However, our original study only included sales data up to September 2020 and was updated during the review process as more recent data became available to the study team.”**

Please remove the information on funding and competing interests from the end of the main text. In the event of publication, this information will appear in the article metadata via entries in the submission form.
**Response: Made changes as suggested.**

Throughout the text, please remove spaces from within the reference call-outs (e.g., "... post-exposure prophylaxis [13,14].").
**Response: Made changes as suggested.**

In the reference list, please use the abbreviation "PLoS ONE".

**Response: Made changes as suggested.**

Noting references 36 and 57, can some additional access information, or a URL, be added?
**Response: Made changes as suggested.**

Please add "[preprint]" to reference 53 and any other cited preprints, and correct the mis-formatting.

**Response: Replaced with published version of the article that has recently become available.**

Please add a completed checklist for the most appropriate reporting guideline, e.g., STROBE, as a supplementary document. This should be labelled "S1_STROBE_Checklist" or similar and referred to as such in your Methods section. In the checklist, please refer to individual items by section (e.g., "Methods") and paragraph number, not line or page numbers (as the latter generally change in the event of publication).
**Response: We have filled out the STROBE Checklist although most fields are not applicable to our study. To our knowledge, no validated checklist is available for the reporting of impact evaluation studies such as ours. For this reason, we leave to the Editors the final decision on whether to keep the STROBE checklist or not.**

**Comments from Reviewers:**

*** Reviewer #2:
Thanks for the revised manuscript and response to my original queries. Figures 1 and 2 are both improved and clear to read. The additional information on IQVIA was helpful - the IQVIA pharmacy data does seem to be widely used and legitimate.
The limitation with the data is that without information about the proprietary method that is used to estimate national data from the sample panel of sellers there is the possibility that this method becomes biased under unusual conditions, e.g. COVID-19. This is acknowledged with respect to changes in lag-time, I think that a general acknowledgement of the limitation of using a propriety estimation method, along with the specific issue of lag time in the discussion would be a sufficient clarification of the issue.

**Response: We thank the reviewer for this comment. We have included the following sentence in the discussion: “Fifth, while data from IQVIA are widely utilized to evaluate pharmaceutical sales, it should be highlighted that the company adopts a propriety method for estimating national sales.”**

*** Reviewer #3:
[supportive report received]

**Response: We thank the reviewer**
